# NHC-CDI Betaine Adducts and Their Cationic Derivatives as Catalyst Precursors for Dichloromethane Valorization

**DOI:** 10.1021/acs.joc.1c01971

**Published:** 2021-11-01

**Authors:** David Sánchez-Roa, Marta E. G. Mosquera, Juan Cámpora

**Affiliations:** † Departamento de Química Orgánica y Química Inorgánica, Instituto de Investigación en Química “Andrés M. del Río” (IQAR) Universidad de Alcalá, Campus Universitario, Alcala de Henares, Madrid 28871, Spain; ‡ Instituto de Investigaciones Químicas, CSIC-Universidad de Sevilla, C/Américo Vespucio, 49, Sevilla 41092, Spain

## Abstract

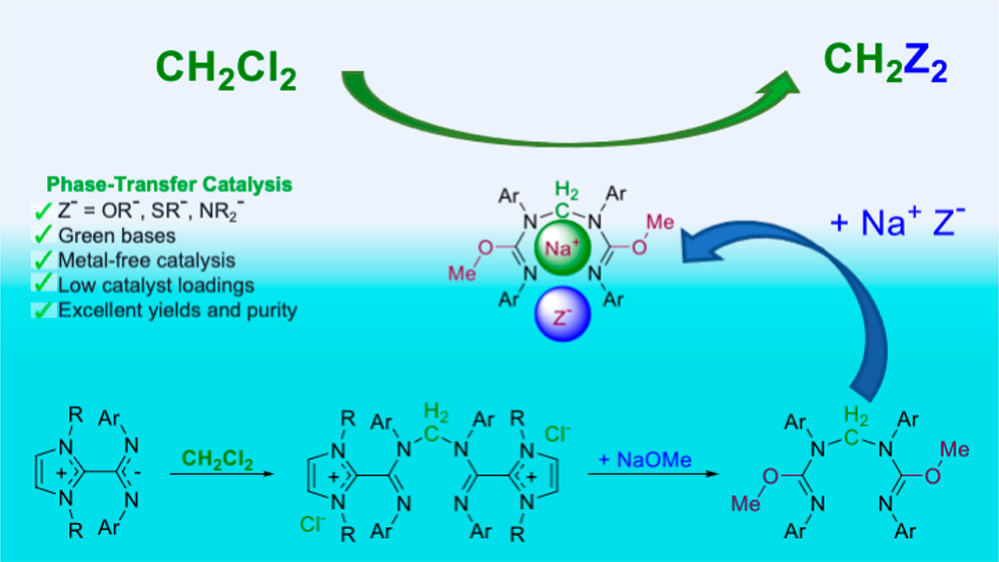

Zwitterionic adducts of N-heterocyclic carbene and carbodiimide (NHC-CDI) are an emerging class of organic compounds with promising properties for applications in various fields. Herein, we report the use of the ICyCDI(*p*-Tol) betaine adduct (**1a**) and its cationic derivatives **2a** and **3a** as catalyst precursors for the dichloromethane valorization via transformation into high added value products CH_2_Z_2_ (Z = OR, SR or NR_2_). This process implies selective chloride substitution of dichloromethane by a range of nucleophiles Na^+^Z^–^ (preformed or generated *in situ* from HZ and an inorganic base) to yield formaldehyde-derived acetals, dithioacetals, or aminals with full selectivity. The reactions are conducted in a multigram-scale under very mild conditions, using dichloromethane both as a reagent and solvent, and very low catalyst loading (0.01 mol %). The CH_2_Z_2_ derivatives were isolated in quantitative yields after filtration and evaporation, which facilitates recycling the dichloromethane excess. Mechanistic studies for the synthesis of methylal CH_2_(OMe)_2_ rule out organocatalysis as being responsible for the CH_2_ transfer, and a phase-transfer catalysis mechanism is proposed instead. Furthermore, we observed that **1a** and **2a** react with NaOMe to form unusual isoureate ethers, which are the actual phase-transfer catalysts, with a strong preference for sodium over other alkali metal nucleophiles.

## Introduction

Dichloromethane (DCM) is a relatively inert compound that is widely applied as a solvent in organic synthesis and separation procedures. It can be regarded as a low polarity, nonprotic, and noncoordinating solvent, which, however, dissolves many polar or even ionic species.^1^ The heavier dihalomethane congeners, dibromomethane and diiodomethane, are considerably more reactive than DCM. Those are rarely used as a solvent but often employed as a methylene source via nucleophilic substitution reactions.^2^ Indeed, along with formaldehyde, dihalomethanes represent an important C1 chemical building block.^3^ DCM could be a convenient, readily available, and inexpensive alternative to these compounds; nevertheless, it is seldom used as a chemical reagent. The chemical stability of C–Cl bonds, which makes DCM such an excellent solvent, usually leads to difficult chloride substitution processes.^4^ The latter are often plagued by side reactions, such as the HCl elimination whenever the nucleophiles behave as strong bases or free radical chain processes.^4a,5^ These unwanted reactions can entail explosion hazards in some cases.^6^ Yet, the use of DCM as the source of the CH_2_ fragment could help improve many chemical transformations beyond the mere cost reduction. Among other advantages, DCM allows much safer handling than formaldehyde, a widely used CH_2_ precursor in classic organic synthesis.^7^ Although the intensive usage of organohalogens can lead to environmental issues, there are strategies that can be followed in order to optimize their application and minimize this impact.^8^ For instance, DCM can be used simultaneously as solvent and reagent, and due to its low boiling point and its poor miscibility with water and aqueous mixtures, it can be readily separated from polar or heavier substances and recycled for further uses.^9^ Among others, clean chlorine substitution in DCM could lead to significant improvements in the syntheses of a wide variety of methylene-bridged derivatives, such as formaldehyde-derived acetals, dithioacetals, or aminals. Formaldehyde acetals or formals are chemicals with many practical applications. Those have proven useful as fuels or fuel additives,^10^ and also as reagents for the introduction of an alkoxy- or aryloxymethylene group.^11^ On the other hand, dithioacetals and aminals are less used in the industry than formals but have important applications in synthesis^12^ and coordination chemistry.^13^ Although transition metal catalysts^14^ (e.g., Ni^14a^ or Cu^14b^ complexes) have been occasionally used for coupling dichloromethane with nucleophiles, the usefulness of such processes is usually limited by the need for high metal loadings and relatively low yields.

In this contribution, we disclose a practical application of stable adducts of NHC carbenes and carbodiimides (NHC-CDI) as suitable catalysts for nucleophilic chloride substitution on DCM, providing a convenient route for a variety of symmetrical, methylene-bridged derivatives CH_2_Z_2_. NHC-CDI’s are an emerging class of dipolar compounds that belong to the chemical class of betaines, electroneutral zwitterions that cannot be represented by any resonance form with full charge cancellation.^15^ NHC-CDI’s bearing aryl substituents in the CDI part are readily prepared and exhibit enhanced stability even under the open air, despite the strongly basic and nucleophilic character of their amidinate moiety.^16^ Due to these properties, stable NHC-CDI adducts have a promising potential for application in many different fields, including metal ligands in coordination and organometallic chemistry,^17^ nanocatalyst design,^18^ building blocks for polymers,^19^ or in the development of new types of persistent free radicals.^20^

**Scheme 1:**
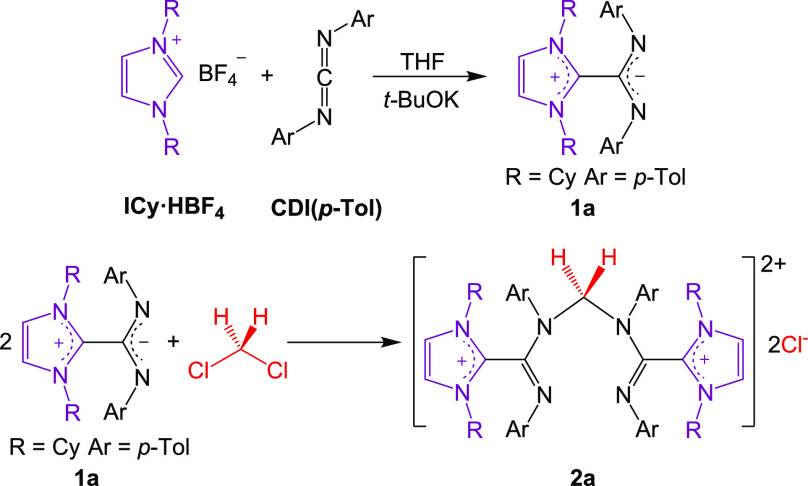
Synthesis of **1a** and **2a**

Recently, we have reported that the ICyCDI(*p*-Tol) betaine adduct (**1a**), an NHC-CDI derivative containing 1,3-dicyclohexylimidazolylidene (ICy) and di-*p*-tolylcarbodiimide (CDI(*p*-Tol)) as the NHC and CDI parts, respectively, reacts in DCM solution to afford the ionic salt [CH_2_(**1a**)_2_]^2+^[2Cl^**–**^] (**2a**·2Cl). Despite the considerable steric bulk of **1a**, this process, which implies the cleavage of both C–Cl bonds of DCM, proceeds in quantitative yield and full selectivity, without any detectable byproducts or intermediates (Scheme 1).^21^ Compound **2a** can be envisaged as a “[CH_2_]^2+^” fragment trapped by two neutral **1a** units. We were intrigued by the possibility that the central core of the **2a** dication could be activated for nucleophilic substitution, delivering this group to mild nucleophiles. In a catalytic version of this process, DCM could be applied as a source of the [CH_2_]^2+^ synthon, and **1a** (or some other related NHC-CDI derivatives) might act as an organocatalyst in an unusual class of organocatalyzed nucleophilic substitution reaction. Herein we show that this is indeed the case, although further investigation of the details shows that the mechanism of this catalytic transformation differs largely from our initial intuition.

## Results and Discussion

**Scheme 2:**
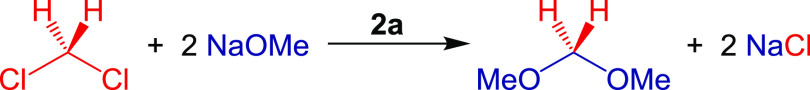
Synthesis of Methylal Using **2a** as a Catalyst

To test our proposal, we first used solid sodium hydroxide as a nucleophile. This reaction led to the substitution of Cl for OH, affording hydrated formaldehyde oligomers, which could be detected in the ^1^H NMR spectra of the mixture^22^ (broad signal at 4.88 ppm, see Figure S25) and also because of the characteristic smell of free formaldehyde, but the efficiency of the reaction was hard to quantify. Therefore, we shifted our trial to solid sodium methoxide, which would enable the synthesis of methylal through the substitution of chlorine atoms of DCM by methoxy groups (Scheme 2). Methylal or dimethoxymethane is an interesting target since it can be used as a green solvent^23^ (far less toxic than DCM itself) or as a safer surrogate of formaldehyde, preventing the use of this toxic substance or its insoluble polymer, paraformaldehyde.^24^ Yet, it has been known for a very old time that neat dichloromethane does not react with sodium methoxide, or it does only reluctantly under forcing conditions to afford complex mixtures of products.^25^ We performed a series of tests with different amounts of solid sodium methoxide suspended in neat DCM at 25 and 60 °C, using a sealed ampule with a magnetic stirrer as a reactor. We confirmed that, whereas hardly any detectable quantity of methylal was formed in the absence of **2a**, the addition of a catalytic amount of the latter (from 2 to 0.2 mol %) led to the quantitative conversion of NaOMe into methylal and NaCl. Due to the similar boiling points of DCM and methylal, we made no attempt to separate the product from the excess solvent. However, spectroscopic analyses of the crude mixtures showed that the transformation is very clean.^26^

The NMR spectrum of a drop of the liquid phase in CDCl_3_ displayed only major signals for methylal (3.30 and 4.52 ppm) and unreacted DCM (5.32 ppm), along with very minor signals from the catalyst. We then decided to scale-up the process to show the potential of this reaction. Hence, the synthesis of methylal was carried out starting from 2 g of sodium methoxide and 20 mL of DCM, at 60 °C, using 0.2 mol % of catalyst **2a**, and full conversion was achieved. Finally, the catalyst loading was decreased to 0.01 mol % (100 ppm) and NaOMe quantity scaled up to 20 g, leading to an apparent TON figure in the order of 10^4^ without any loss of purity or selectivity. The use of such a small proportion of a metal-free catalyst is remarkable.^27^

**Figure 1:**
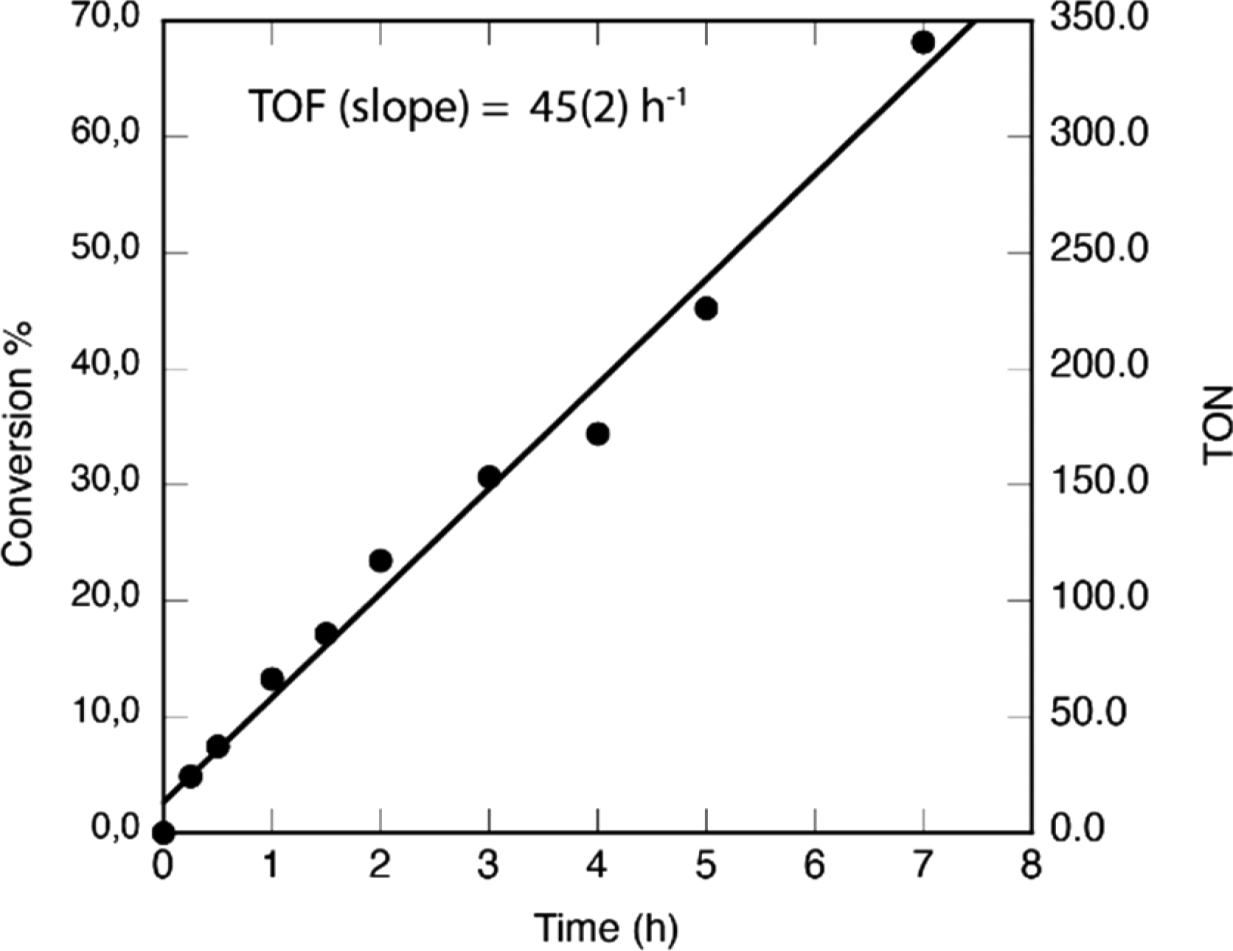
Yield vs time plot for the reaction of DCM with NaOMe catalyzed by **2a**. Conditions: 40 °C, NaOMe, 2 g (31 mmol); DCM (neat) 40 mL (626 mmol); **2a**, 37 mg (0.2 mol % with regard to NaOMe). The last check after 24 h was consistent with full NaOMe consumption.

NMR analyses of samples removed at regular intervals from a stirred suspension of solid NaOMe in DCM containing **2a** (0.2 mol % vs NaOMe) at 40 °C showed that the methylal content of samples increased linearly up to 70% conversion (68% within 7 h) and was consistent with full conversion when a final aliquot was measured after 24 h (Figure 1). These data indicate that the activity of **2a** does not show any significant decay over long periods of time, at a turnover frequency (TOF) of 45 h^–1^ (referred to DCM, or 90 h^–1^ for NaOMe) under such mild conditions. Moreover, the addition of a second 2 g portion of sodium methoxide to a completed reaction mixture containing an excess of unreacted DCM led again to full conversion within the expected 24 h period, suggesting that this catalyst remains indefinitely active under the specified conditions.

With this first accomplishment in hand, we set out to explore other suitable nucleophiles to define the scope of this interesting reaction. In particular, we analyzed whether other O, S, or N-based nucleophiles would perform in a similar way in order to obtain formaldehyde-derived acetals, dithioacetals, or aminals, respectively. The results are displayed in Table 1. We first explored the reaction of DCM with solid sodium phenoxide and sodium benzyloxide under the same conditions developed for the synthesis of methylal (entries 2 and 3). We selected these alkoxides because they should exhibit milder reactivity than sodium methoxide. Yet, both of them reacted with DCM under mild conditions, affording the corresponding products in a highly selective manner. Essentially pure products were isolated after a very simple workup involving filtration through a silica pad (to remove NaCl and the remaining catalyst) and evaporation under a vacuum. The purity and quantitative isolated yield confirmed the high selectivity appreciated in the NMR analyses of the crude mixtures.

**Table 1:** Catalyzed Nucleophilic Substitution Reactions in Dichloromethane with Various Nucleophiles and Conditions^a^

entry	product CH_2_Z_2_^b^	substrate/base^c^	cat.	conversion^d^/yield^e^
1	i	NaOMe	**2a**	>99/>99
2	ii	NaOPh	**2a**	>99/92
3	iii	NaOBn	**2a**	>99/87
4	i	MeOH/NaOH	**2a**	>99/89
5	ii	PhOH/NaOH	**2a**	>99/89
6	iii	BnOH/NaOH	**2a**	>99/86
7	iv	PTBP^f^/NaOH	**2a**	>99/85
8	v	AA^g^/NaOH	**2a**	>99/83
9	vi	*i-*PrOH/NaH	**2a**	74/67
10	vii	EtSH/NaOH	**2a**	>99
11	viii	HPz^h^/NaH	**2a**	89/72
12	i	NaOMe	**1a**	>99/>99
13	ii	NaOPh	**1a**	>99/86
14	iii	NaOBn	**1a**	>99/87
15	i	NaOMe	**3a**	>99/>99

a Reaction conditions: catalyst loading 0.2 mol %, 60 °C, 24 h.

b See Scheme 3 for the detailed structure of the products.

c Insoluble bases (NaOH, NaH), added in excess.

d Spectroscopic yield from ^1^H NMR.

e Isolated yields (calculated on the basis of the starting substrate), unless otherwise specified.

f *p*-*tert*-Butylphenol.

g Allyl alcohol.

h Pyrazole.

In view of the excellent results of transformations with solid alkoxides, we examined a more practical setup for this catalytic reaction (Scheme 3). Thus, methylal was obtained using methanol combined with solid sodium hydroxide, a much cheaper and safer base than NaOMe. Although the spectroscopic yield of this reaction was slightly lower, the selectivity was excellent again since methylal was the only organic product detected, along with unreacted DCM. Despite the fact that **2a** also catalyzes the reaction of DCM with NaOH, no traces of formaldehyde or formaldehyde oligomers were detected in the NMR spectra of the reaction mixture. The apparent losses in the mass balance can be attributed to the poor mechanical properties of the sticky solid formed by wet sodium salts, which could occlude some methanol.

**Scheme 3:**
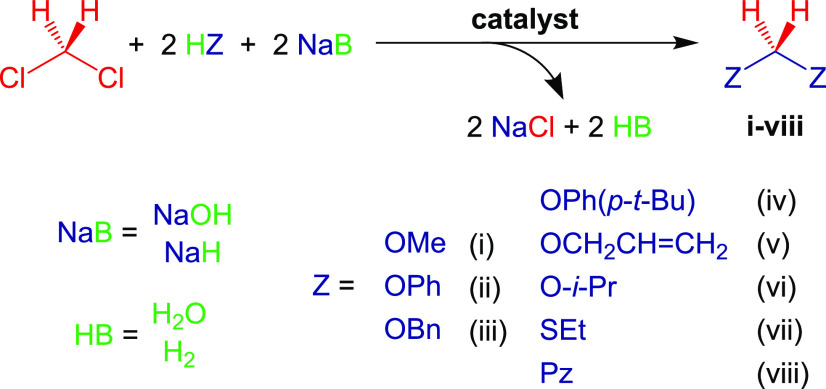
Catalytic Transformation of DCM into CH_2_Z_2_ Using Combinations of Weak Protic Acids and Suitable Bases (NaOH or NaH)

In a similar fashion, **2a** efficiently catalyzed the reaction of several alcohols with DCM at 0.2 mol % catalyst loading in the presence of suitable bases. Among the substrates, those that gave the best conversions with NaOH were primary alcohols and phenol derivatives. Thus, benzyl alcohol, allyl alcohol (AA), phenol, and *p*-*tert*-butylphenol (PTBP), combined with NaOH, were cleanly transformed into the corresponding acetals with perfect selectivity and excellent yields. NaOH was not effective for 2-propanol, a less acidic secondary alcohol. Nevertheless, when the stronger base NaH was used, the corresponding acetal was produced. Surprisingly enough, the acidic *p*-chlorophenol turned out to be unreactive, either in the presence of NaOH or as the solid sodium salt, which we attribute to the low nucleophilicity of the corresponding anion. Regarding the synthesis of dithioacetals, we used ethanethiol as a reference for thiols, which was successfully converted to di(ethylthio)methane using NaOH as a base. For the synthesis of aminals, we performed the synthesis of bis(pyrazolyl)methane. This target was selected in view of the wide application of pyrazolyl-based molecules as chelating ligands for metal complexes.^13^ Also, in this case, the functionalization of both C–Cl bonds of DCM and the subsequent formation of new C–N was achieved, but NaH was required for the deprotonation of pyrazole (HPz).

### Mechanistic Studies

**Scheme 4:**
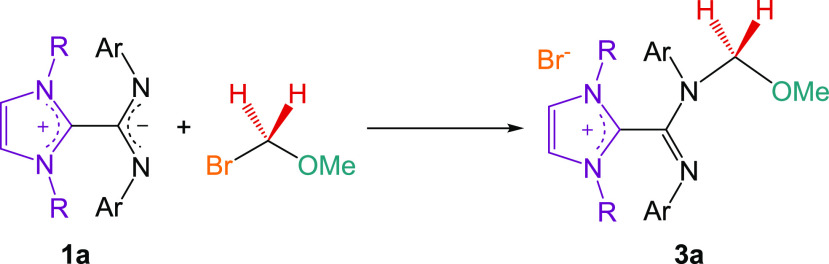
Synthesis of the Catalyst Precursor **3a**

According to our initial ideas, the role of dication **2a** would be to act as a source of the [CH_2_]^2+^ synthon, regenerating the precursor betaine **1a** in each turn of the catalytic cycle. Accordingly, not only **2a** but also betaine **1a** would be active catalysts for this reaction. Similar reasoning led us to conclude that cationic derivatives, [ZCH_2_·**1a**]^+^, would participate as short-lived intermediates in the catalytic cycle, preceding the introduction of the second Z fragment. To test these hypotheses, we synthesized the salt [MeOCH_2_·**1a**]^+^[Br^–^] (**3a**) as shown in Scheme 4 and compared its catalytic performance with those of **1a** and **2a** in the synthesis of methylal. Gratifyingly, as can be seen in Table 1, all three compounds perform as catalysts in the synthesis of methylal, with similar efficiency. Moreover, **1a** was tested in the synthesis of diphenoxymethane and dibenzyloxymethane with identical results as **2a** (Table 1, entries 12–15). However, we realized that the TON and TOF figures of our system were atypical for a regular organocatalytic mechanism involving systematic C–N bond formation and cleavage. On the basis of these data and previous qualitative observations,^21^ it can be easily shown that this reaction is at least 2–3 orders of magnitude faster than the reaction of **1a** with DCM at the same temperature,^28^ which rules out our preliminary proposals.

**Figure 2:**
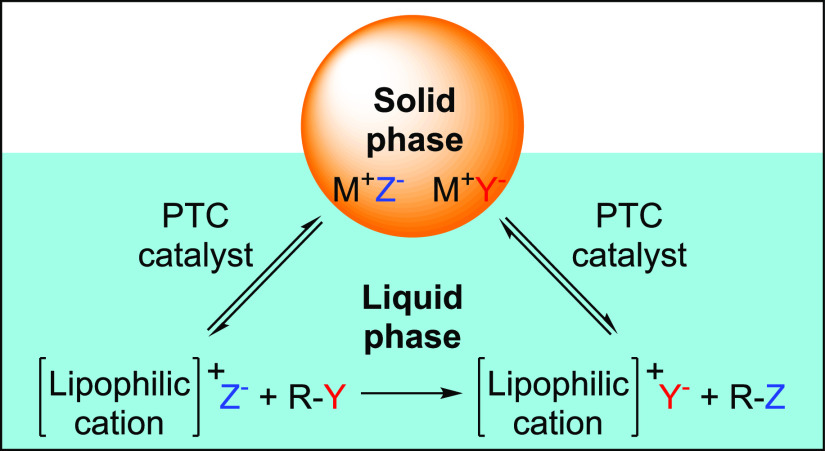
Solid–liquid phase-transfer catalysis.

An alternative phenomenon that would account for the high activity levels observed in our system is solid–liquid phase-transfer catalysis (PTC). PTC allows the solubilization of highly polar or ionic species in nonpolar solvents. Insoluble reagents that normally would be forced to react through the solid–liquid interphase are transported into the solution, where they react under rather diluted conditions. Therefore, solid–liquid PTC enhances the reactivity of solids, and at the same time improves selectivity, by reducing side processes that are common when highly reactive species are dissolved in a higher concentration (Figure 2).^29^ Solid–liquid PTC is often regarded as a green methodology, as reagents and saline products are kept in the solid state and can be readily separated by physical methods.^30^ Alkylation of alcohols and other nucleophilic substrates under PTC conditions is not a novel procedure, but the potential of this methodology using DCM both as a solvent and as alkylating reagent remains almost unexplored.^31^ Although DCM has been found an excellent solvent for liquid–solid PTC,^32^ in the rare cases when DCM is used as an electrophile, liquid–liquid PTC methods in biphasic media (with concentrated aqueous NaOH), or highly polar solvents like *N*-methyl-2-pyrrolidone have been preferred.^33^ Interestingly, the above-mentioned transition metal catalysts also used DCM both as electrophile and solvent.^14^

Several considerations point to PTC as the mechanism that best explains the high activity of our catalysts. First of all, a process controlled by the phase transfer step is consistent with the linear kinetic plot shown in Figure 1, which indicates a zero-order dependency on the reagents well over 50% conversion of NaOMe. Second, to put our results in the correct perspective, we decided to compare the performance of **1a**, **2a**, or **3a** with those of a set of typical phase-transfer agents, like onium-type salts and crown ethers for the synthesis of methylal under the same experimental conditions (Table 2). To enable a more accurate comparison, we ran the experiments under the conditions required by **2a** (0.2 mol %) to drive the reaction of DCM with NaOMe to ca. 50% conversion. The only difference with the preparative conditions was that, in order to facilitate data collection, this set of experiments was performed at a slightly lower temperature, 40 °C, at which the half-conversion time would be ca. 4 h. As shown in Table 2, no methylal was formed in the absence of any PTC reagent, confirming the inertness of DCM against solid NaOMe reported in old literature sources cited above.^25^ Nevertheless, when we added the same catalyst loading of NBu_4_Cl, 18-crown-6, 15-crown-5, or the imidazolium salt ICy·HBF_4_ (the precursor of the NHC carbene ICy, one of the constitutive parts of **1a**), DCM conversion was observed, albeit with significant differences. Remarkably, **2a** proved the most active of the set. NBu_4_Cl, one of the best-known and widely used PTC reagents, is also active, with slightly lower activity, and the imidazolium salt showed a rather poor conversion. Moreover, we tested whether CDI(*p*-Tol), the carbodiimide moiety of **1a**, was also active, but no conversion was achieved. At any rate, the efficiency of both **1a** and **2a** are comparable even to NBu_4_Cl and are clearly superior to a typical crown ether, such as 18-crown-6.

**Table 2:** Catalytic studies of **1a**, **2a**, **3a**, and Other PTC Catalysts toward MOMe (M = Li, Na, K)

entry	substrate	catalyst^a^	conversion^b^ (%)
1	NaOMe		
2	NaOMe	NBu_4_Cl	44
3	NaOMe	18-crown-6	20
4	NaOMe	15-crown-5	39
5	NaOMe	ICy·HBF_4_	8
6	NaOMe	CDI(*p*-Tol)	
7	NaOMe	**1a**	46
8	NaOMe	**3a**	43
9	NaOMe	**2a**	55
10	LiOMe	**2a**	
11	KOMe	**2a**	5

a Catalyst or catalyst precursor (see below).

b An aliquot was taken after 4 h to calculate conversion by ^1^H NMR.

The mechanism responsible for the solubilization of insoluble nucleophiles depends largely on the nature of the PTC catalyst. Whereas tetraalkylammonium salts, like NBu_4_Cl, rely on the high solubility of its cation in low polarity solvents, the action of 18-crown-6 is due to the sequestration of the alkali metal cation in the cavity of the molecule.^29,30^ Hence, the lower efficacy of 18-crown-6 in the reaction with NaOMe could be due to its moderate affinity for Na^+^ (e.g., the affinity ratio K^+^/Na^+^ for 18-crown-6 in the gas phase is 10:6).^34^ It is well-known that the efficacy of complexing PTCs shows significant dependency on the size of the alkali metal.^35^ As shown in entry 4, 15-crown-5, whose smaller cavity is best suited for Na^+^, performs better than 18-crown-6, but it is still inferior to **2a**. Similarly, compound **2a** shows a strong preference for Na^+^, performing its action in a much more efficient manner with NaOMe than with KOMe, whereas LiOMe turned out to be ineffective (Table 2, entries 9–11). These observations suggest that, in addition to the cation exchange solubilization mechanism, our catalysts may simultaneously be performing as selective ligands for the alkali metal. This conclusion is a somewhat unexpected result since the positive charge of the betaine derivatives (double in the case of **2a**) should reduce their capacity to coordinate to cationic alkali metal centers and, in consequence, would be expected to behave in a nonspecific manner, like quaternary ammonium salts.

**Scheme 5:**
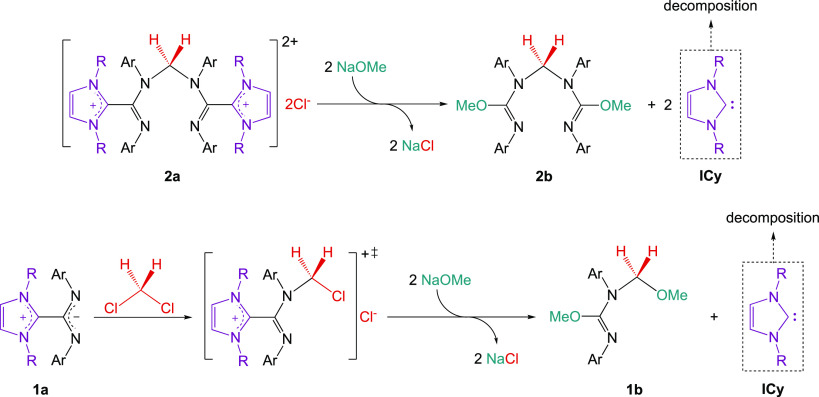
Transformation of **2a** and **1a** into **2b** and **1b**, Respectively, in Catalytic Media

A significant detail observed during the reaction of NaOMe and DCM catalyzed by either **1a** or the cations **2a** and **3a** is the evident intensification of the color within the initial hours of the reactions, leaving a strong reddish-orange tone that persists once the reaction is finished. Strongly colored reaction mixtures were also observed with other nucleophiles, independently on whether **1a** or **2a** were used as catalysts, but, revealingly, no color change was observed when the catalysis was unsuccessful, as in the reactions with *p*-chlorophenoxide or with lithium methoxide. In an attempt to identify the colored materials formed with NaOMe, we recorded the ^1^H and ^13^C{^1^H} spectra of the deeply colored oils remaining after evaporation of the reaction mixtures catalyzed with either **1a** or **2a**, by dissolving in CD_2_Cl_2_. The NMR spectra of the residue left by **2a** was particularly clean, showing that the original signals of the methylene-bridged dication had fully disappeared and replaced with a new spectrum of what looked like a mixture containing a strongly prevalent species **2b** (see Figure S20). The DOSY spectrum of the mixture (see Figure S19) allows a clear distinction of the signals of the main product, **2b**, from those of background species, which have all significantly higher diffusion coefficient or, what is the same, lower molecular weight. The spectra of **2b** correspond to a symmetrical species akin to **2a**, which has no imidazolium moieties. The broad and ill-defined signals of the cyclohexyl fragments are all associated with the low molecular weight region, consistent with the degradation of the ICy unit under the reaction conditions. Concerning **2b**, a single resonance of relative intensity for 6H is found at 3.53 ppm, corresponding to two equivalent methoxy groups, along with another singlet of 2H for the central CH_2_ unit at 5.15 ppm. The relative simplicity of the spectra of **2b** enabled us to fully assign by ^1^H and ^13^C NMR the unusual isoureate ether structure shown in Scheme 5. The NMR spectra of the residue obtained from the **1a**-catalyzed reaction (see Figure S17) indicates the presence of the prevalent species, **1b**, different from the starting betaine and from **2b**, which contains two resonances MeO (3.30 and 3.75 ppm) and a single CH_2_ unit at 4.69 ppm. Interestingly, the DOSY spectrum of this mixture (see Figure S16) gave slightly lower size to the molecules of **1b** than to the broad background arising from the imidazolium fragment. These data clearly point to the unprecedented processes shown in Scheme 5, giving rise to **1b** and **2b**, rare examples of isoureate ethers, which are likely responsible for most of the PTC activity in the catalytic reaction. Accordingly, ESI-MS of partially purified extracts in wet MeOH showed intense signals for the corresponding monoprotonated ions (*m*/*z* = 299 and 521, respectively), as well as their corresponding Na^+^ complexes (321 and 543). Ion compositions were confirmed in the corresponding HR-ESI spectra (see Experimental part). Note that **1b** is the result of trapping the reactive chloromethyl intermediate [ClCH_2_·**1a**]^+^[Cl^–^] by a methoxide, giving the same characteristic methoxymethylene fragment, also present in **3a**. This was confirmed by comparing the NMR spectra of **3a** with the crude residue left after evaporation of DCM/methylal solutions (see Figure S24). Thus, **1b** cannot be converted in **2b** during the reaction. In consequence (and in contrast with our initial expectations), each of the catalytic reactions initiated by precursors **1a** and **2a** are due to a different catalytic species. According to this proposal, the catalyst generated from **3a** is, very likely the same formed from **1a**, namely, **1b**.

Attempts to separate **1b** and **2b** from the mixture of products resulting from the degradation of the NHC units have been unsuccessful so far, apparently due to the sensitivity of the isoureate ethers, which seem to hydrolyze on attempted purification by typical chromatographic workup. However, since the imidazolium precursor ICy·HBF_4_ shows very poor activity as a PTC catalyst, the effectivity of both **1a** and **2a** should be entirely ascribed to the electroneutral isoureate ethers, that, as commented above, must have a significant affinity for the Na^+^ cation. This is in accordance with the facile ionization of the neutral molecules with traces of Na^+^ in the methanol solvent. The low catalytic activity found for the imidazolium salt is consistent with these observations, as this is expected to be deprotonated to the same ICy moiety subsequently degrades under the reaction conditions. Likewise, the experiment performed with CDI(*p*-Tol) as a catalyst (Table 2, entry 6) demonstrates not only that the free carbodiimide is not be involved in the process, but also that the active isoureate **2b** cannot be generated directly by the successive reaction of CDI(*p*-Tol) with NaOMe and DCM. To confirm this point, we attempted the reaction of CDI(*p*-Tol) with NaOMe in DCM but, as expected, no reaction was observed. Thus, the only plausible pathway to generate the catalytically active species **1b** and **2b** is through betaine **1a** and its cationic derivatives **2a** and **3a**.

## Conclusions

In summary, we have shown that efficient catalytic systems generated from betaine **1a** or their cationic alkyl derivatives **2a** and **3a** provide a versatile and clean method for the conversion of DCM in a range of formaldehyde derivatives CH_2_Z_2_. We have successfully applied this transformation to aliphatic and aromatic alcohols, thiols, and N-heterocycles, which can be used either as insoluble sodium salts or directly, in combination with suitable bases like NaOH or NaH, depending on their acid strengths. This process only generates sodium chloride and water or hydrogen as byproducts, whose straightforward elimination greatly facilitates the purification of the products. Furthermore, DCM transformation was achieved at very low catalyst loadings and required no transition metals. The volatility of dichloromethane allows its facile recovery and recirculation, enabling facile scale-up of the reactions. Mechanistic studies have ruled out an organocatalytic cycle involving the reversible formation and cleavage of C–N bonds of **2a**, which rather acts as a precursor for electroneutral, highly efficient Na^+^ complexing isoureate ethers arising from an unusual nucleophilic displacement of the ICy (the NHC fragment of the betaine) by the nucleophilic reagent. We are currently extending our studies to other NHC-CDI derivatives and investigating the potential uses of these compounds in catalysis, either free or complexed to abundant and low-toxicity metals.

## Experimental Section

### General Considerations

All manipulations were performed under an inert atmosphere using Schlenk-line techniques (O_2_ < 3 ppm) and a glovebox (O_2_ < 0.6 ppm) MBraun MB-20G. Solvents were purified using an MBraun Solvent Purification System, except dichloromethane (DCM) and THF, which were distilled with CaH_2_ and Na, respectively. All NMR scale experiments were carried out in sample tubes with airtight PTFE valves. Deuterated solvents were degassed and stored in the glovebox in the presence of molecular sieves (4 Å). NMR spectra were recorded with a Bruker 400 Ultrashield (^1^H 400 MHz, ^13^C 101 MHz) at 25 °C. All chemical shifts were determined using residual signals of solvents and were referenced with regard to external SiMe_4_. Assignments of spectral signals were helped with 2D (^1^H–^13^C HSQC and HMBC) and diffusion (DOSY) NMR experiments. Elemental analysis and ESI-MS spectra of samples were carried out by the Analytical Services of the Institute for Chemical Research (Seville, Spain) using an LECO CHNS-TruSpec and Bruker Ion Trap Bruker Esquire 6000, respectively. HR-ESI spectra were recorded by the Mass Spectrometry Service (CITIUS, University of Seville) in a Thermo Scientific Orbitrap Elite hybrid mass spectrometer, operating in direct injection mode with an ESI ion source and ion-trap analyzer. Unless otherwise specified, all commercial reagents were purchased from Sigma-Aldrich and used as received. Imidazolium salt ICy·HBF_4_ and catalysts **1a** and **2a** were prepared according to literature and our synthetic procedures, previously reported.^17a,21,36^ Warning! Although dichloromethane does not react vigorously with NaH, NaOH, or sodium alkoxides or aryloxides described in this work, strongly basic reagents like potassium *tert*-butoxide or neat sodium, which are known to react violently with this solvent, should be avoided.

### General Procedure

A regular magnetic bar sufficed to achieve a satisfactory mixing of the suspensions throughout the whole experiment. DCM, containing the required amount of catalyst, was added to a gastight glass Teflon valve glass ampule containing solid Na^+^Z^–^ (either preformed or generated *in situ* from HZ and an inorganic base), and the mixture was stirred for a prescribed time in an oil bath preset at the specified temperature. In all experiments, the mixture gradually takes an intense yellow-orange color, as the suspended solid gradually becomes a thinner precipitate (NaCl). At the prescribed time, the mixture was allowed to settle, and a small sample (0.1–0.05 mL) was taken and dissolved in CDCl_3_. The conversion was deduced from the relative ratios of the central CH_2_ signal of the product and an internal standard (hexamethylbenzene). See below for more details and the purification procedures.

### Preliminary Experiments

With NaOH, an excess (1 g) of NaOH was added to a vial, and 10 mL of a solution of 20 mg of catalyst **2a** in neat dichloromethane was added to the solid. After 24 h, a free formaldehyde smell was detected, and an aliquot of the mixture was taken. The NMR spectrum of the mixture in CDCl_3_ showed a broad signal for hydrated formaldehyde oligomers (see Figure S25), but the efficiency of the reaction was hard to quantify.

With NaOMe in an NMR tube, catalyst **2a** (5 mg, 2 mol %) was dissolved in CD_2_Cl_2_, and then anhydrous NaOMe (27 mg) was added inside the NMR tube. It was not soluble and remained in the bottom of the tube. After 2 h at room temperature, the solution became light orange, and it got more intense over time. It was also perceived a change in the texture of the solid, which became thinner. The ^1^H NMR spectrum showed an intense singlet at 3.30 ppm, which was assigned to 1H of methoxy groups from dimethoxymethane (methylal). As the methylene source was CD_2_Cl_2_, the methylene fragment of the product was not observed.

### Optimization of the General Reaction Conditions for the Syntheses of Methylal from DCM and NaOMe with Catalyst **2a**

In the general procedure (see Table S1 for details), a regular magnetic bar sufficed to achieve a satisfactory mixing of the suspensions throughout the whole experiment. DCM, containing the required amount of **2a**, was added to a Schlenk flask containing solid NaOMe, and the mixture was stirred for a prescribed time. In the experiments conducted at 60 °C, the mixture was transferred to a gastight glass Teflon valve glass ampule, which was closed and magnetically stirred in an oil bath preset at the specified temperature. In all experiments, the mixture gradually takes an intense yellow-orange color, as the suspended solid gradually becomes a thinner precipitate (NaCl). At the prescribed time, the mixture was allowed to settle, and a small sample (0.1–0.05 mL) was taken and dissolved in CDCl_3_. The conversion was deduced from the relative ratios of the CH_2_ signals of unreacted dichloromethane and methylal. The mixture was then allowed to settle, filtered with a cannula, and vacuum-transferred to a cold trap, which affords a clean, colorless solution of methylal in the remaining dichloromethane. The mixture was a colorless liquid. ^1^H NMR (CDCl_3_, 400 MHz): δ 4.52 (s, 2H), 3.30 (s, 6H). ^13^C{^1^H} NMR (CDCl_3_, 100 MHz): δ 97.7, 55.2. Spectroscopic data is in accordance with data reported in literature.^38a^

### Monitoring the Reaction of NaOMe with CH_2_Cl_2_

A 50 mL glass reactor provided with a Young Teflon screw stopcock and a nitrogen-purged liner to allow sample removal was charged with solid NaOMe (2 g, 37 mmol), DCM (35 mL), and 5 mL of a DCM solution containing 37 mg of catalyst **2a** (0.2 mol %), and an accurately weighed amount (30 mg) of hexamethylbenzene to be used as an internal standard, weigh to ±0.1 mg accuracy in an analytical balance. The mixture was stirred at 40 °C in an oil bath preset at the specified temperature, taking aliquots of ca. 0.1 mL with a long-needle syringe through the sample removal port. Each sample was diluted with 0.5 mL of CDCl_3_, filtered, and analyzed using NMR to determine the reaction advance. The NMR data were positively compared with those listed in the SDBS spectral database in the same solvent (CD_3_Cl).^38a^

### Comparative Assessment of Catalyst Efficiency in the Methylal Synthesis Using Different Catalysts and Alkali Metal Cations

In order to study the effect of the catalyst in the obtention of methylal, we repeated the reaction under the same conditions described in the previous section, using the same molar amount of a different catalyst in each of them: NBu_4_Cl, 18-crown-6, 15-crown-5, **1a**, **3a** or ICy·HBF_4_, CDI(*p*-Tol). These reactions were not monitored but stopped after a fixed reaction time (4 h at 40 °C, when 50% conversion NaOMe was estimated using **2a**), and their progress was determined using the same NMR-based methodology to determine the amount of methylal formed. Likewise, the efficiency of **2a** with different alkali metal cations was assessed using equivalent amounts (37 mmol) of the corresponding alkali methoxides MOMe (M = Li, Na, or K) and a 4 h reaction time. The characteristic dark orange color associated with these reactions was not observed when LiOMe was used as the methoxide source.

### Procedure for the Generation of Solid Sodium Benzyloxide and Sodium Phenoxide

Sodium benzyloxide or phenoxide was generated prior to use, following a modified procedure taken from the literature:^37^ a 100 mL Schlenk flask equipped with a stirring bar was charged with NaH (82.5 mmol) in mineral oil and 25 mL of THF. The flask was brought to an ice–water bath, and the solid was suspended with magnetic stirring. A solution containing 75 mmol of the corresponding alcohol or phenol in 25 mL of THF was kept cool with vigorous stirring. The addition caused the evolution of H_2_ gas, more vigorous in the case of phenol. Once the addition was completed, the mixture was kept stirring at room temperature for 4 h. The solvent was removed under a vacuum, and the sticky white solid obtained was washed three times with hexane, in order to remove the mineral oil, to afford a white powder. No further purification was performed.

### Synthesis of CH_2_(OPh)_2_ from Sodium Phenoxide

Sodium phenoxide (3.83 g, 33 mmol), generated from phenol and NaH as described above, was added to an ampule with an airtight PTFE valve and suspended in 15 mL of dried dichloromethane. A solution of 33 mg of catalyst **2a** (0.2 mol %) in 5 mL of dried dichloromethane was added with a syringe directly upon the suspension. The resulting colorless mixture was stirred at 60 °C for 24 h in an oil bath preset at the specified temperature. After a few minutes at 60 °C, an intense yellow-orange color in the solution was observed. Once the reaction finished, the stirring was stopped. A light white solid (NaCl) remained in the bottom of the flask, leaving the colored solution clear to perform the purification. The solution was separated from the solid by filtration via cannula. The mixture was filtrated through a pad of silica gel in order to remove the traces of the catalyst, and afterward, the solvent was removed under a vacuum to afford a slightly pale-yellow liquid with a yield of 92% (3.04 g). The same procedure was conducted with 16.5 mg of **1a** as a catalyst, with a yield of 86% (2.84 g). ^1^H NMR (CDCl_3,_ 400 MHz): δ 7.32 (m, spin system AA′BB′C, 4H), 7.13 (m, spin system *AA′*BB′C, 4H), 7.05 (tt, *J* = 7.4 Hz, *J* = 1.1 Hz, 2H), 5.75 (s, 2H). ^13^C{^1^H} NMR (CDCl_3_, 100 MHz): δ 157.2, 129.7, 122.6, 116.7, 91.4. IR (cm^–1^): 3041 (ν C_Ar_—H), 2973 (ν C_sp3_—H), 1588 (ν C_Ar_=C_Ar_), 1490 (ν C_Ar_=C_Ar_), 1200 (ν_as_ C_Ar_—O-C), 1012 (ν_s_ C_Ar_–O-C). Spectroscopic data is in accordance with data reported in literature.^38b^

### Synthesis of CH_2_(OBn)_2_ from Sodium Benzyloxide

Sodium benzyloxide (2.18 g, 16.5 mmol), generated from benzyl alcohol and NaH as described above, was added to an ampule with an airtight PTFE valve and suspended in 15 mL of dried dichloromethane. A solution of 16.5 mg of catalyst **2a** (0.2 mol %) in 5 mL of dried dichloromethane was added with a syringe directly upon the suspension. The resulting colorless mixture was stirred at 60 °C for 24 h in an oil bath preset at the specified temperature. After a few minutes at 60 °C, an intense orange-red color in the solution was observed. Once the reaction finished, the stirring was stopped. A light solid white solid (NaCl) remained in the bottom of the flask, leaving the colored solution clear to perform the purification. The solution was separated from the solid by filtration via cannula. The mixture was filtrated through a pad of silica gel in order to remove the traces of the catalyst, and afterward, the solvent was removed under a vacuum to afford a slightly pale-yellow liquid, with a yield of 87% (1.64 g). The same procedure was conducted with 8.25 mg of **1a** as a catalyst with a yield of 87% (1.64 g). ^1^H NMR (CDCl_3_,400 MHz): δ 7.37 (m, 8H). 7.32 (m, 2H), 4.86 (s, 2H), 4.67 (s, 4H). ^13^C{^1^H} NMR (100 MHz, CDCl_3_, 25 °C): δ 138.0, 128.5, 128.1, 127.8, 94.1, 69.7. IR (cm^—1^): 3029 (ν C_Ar_—H), 2879 (ν C_sp3_–H), 1497 (ν C_Ar_=C_Ar_), 1102 (ν_as_ C—O—C), 1041 (ν_s_ C—O–C). Spectroscopic data is in accordance with data reported in literature.^38c^

### Synthesis of CH_2_(OMe)_2_ from Methanol and Sodium Hydroxide

Sodium hydroxide (1.7 g, 44.5 mmol) was ground and added to an ampule with an airtight PTFE valve and suspended on 25 mL of dried dichloromethane. Afterward, 1.19 g of methanol (37 mmol) was added to the mixture, which caused the appearance of a white sticky solid, sodium methoxide. A solution of 37 mg of catalyst **2a** (0.2 mol %) in 5 mL of dried dichloromethane was added with a syringe directly upon the suspension. The resulting colorless mixture was stirred at 60 °C for 24 h in an oil bath preset at the specified temperature. The stirring was quite hindered at the beginning due to the formation of a water phase. After a few minutes at 60 °C, an intense yellow-orange color in the solution was observed. Once the reaction finished, the stirring was stopped. The organic layer was separated from the solid by filtration via cannula. The yellow mixture was filtrated through a pad of silica gel in order to remove the traces of the catalyst to afford a colorless solution of CH_2_(OMe_2_)_2_ in dichloromethane with a yield of 89%. ^1^H NMR (CDCl_3_, 400 MHz): δ 4.52 (s, 2H), 3.30 (s, 6H). ^13^C{^1^H} NMR (CDCl_3_, 100 MHz): δ 97.7, 55.2. Spectroscopic data is in accordance with data reported in literature.^38a^

### Synthesis of CH_2_(OPh)_2_ from Phenol and Sodium Hydroxide

Sodium hydroxide (0.37 g, 9.25 mmol) was ground and added to an ampule with an airtight PTFE valve and suspended in 15 mL of dried dichloromethane. Afterward, 0.78 g of phenol (8.25 mmol) was added to the mixture, which caused the appearance of a white sticky solid, sodium phenoxide. A solution of 8.25 mg of catalyst **2a** (0.2 mol %) in 5 mL of dried dichloromethane was added with a syringe directly upon the suspension. The resulting colorless mixture was stirred at 60 °C for 24 h in an oil bath preset at the specified temperature. The stirring was quite hindered at the beginning due to the formation of a water phase. After a few minutes at 60 °C, an intense yellow-orange color in the solution was observed. Once the reaction finished, the stirring was stopped. The organic solution was separated from the solid by filtration via cannula. The mixture was filtrated through a pad of silica gel in order to remove the traces of the catalyst. Afterward, the solvent was removed under a vacuum to afford a white powder in 89% yield (0.74 g). ^1^H NMR (CDCl_3_, 400 MHz): δ 7.32 (m, 4H). 7.13 (m, 4H), 7.05 (tt, *J* = 7.4 Hz, *J* = 1.1 Hz, 2H), 5.75 (s, 2H).^13^C{^1^H} NMR (CDCl_3,_ 100 MHz): δ 157.2, 129.7, 122.6, 116.7, 91.4. IR (cm^–1^): 3041 (ν C_Ar_—H), 2973 (ν C_sp3_—H), 1588 (ν C_Ar_=C_Ar_), 1490 (ν C_Ar_=C_Ar_), 1200 (ν_as_ C_Ar_—O—C), 1012 (ν_s_ C_Ar_—O—C). Spectroscopic data is in accordance with data reported in literature.^38b^

### Synthesis of CH_2_(OBn)_2_ from Benzyl Alcohol and Sodium Hydroxide

Sodium hydroxide (0.37 g, 9.25 mmol) was ground and added to an ampule with an airtight PTFE valve and suspended in 15 mL of dried dichloromethane. Afterward, 0.89 g of benzyl alcohol (8.25 mmol) was added to the mixture, which caused the appearance of a white sticky solid, sodium benzyloxide. A solution of 8.25 mg of catalyst **2a** (0.2 mol %) in 5 mL of dried dichloromethane was added with a syringe directly upon the suspension. The resulting colorless mixture was stirred at 60 °C for 24 h in an oil bath preset at the specified temperature. The stirring was quite hindered at the beginning due to the formation of a water phase. After a few minutes at 60 °C, an intense yellow-orange color in the solution was observed. Once the reaction finished, the stirring was stopped. The organic solution was separated from the solid by filtration via cannula. The mixture was filtrated through a pad of silica gel in order to remove the traces of the catalyst. Afterward, the solvent was removed under a vacuum to afford a white powder in 89% yield (0.84 g). ^1^H NMR (CDCl_3,_ 400 MHz): δ 7.37 (m, 8H), 7.32 (m, 2H), 4.86 (s, 2H), 4.67 (s, 4H). ^13^C{^1^H} NMR (100 MHz, CDCl_3_, 25 °C): δ 138.0, 128.5, 128.1, 127.8, 94.1, 69.7. IR (cm^—1^): 3029 (ν C_Ar_—H), 2879 (ν C_sp3_–H), 1497 (ν C_Ar_=C_Ar_), 1102 (ν_as_ C—O—C), 1041 (ν_s_ C—O–C). Spectroscopic data is in accordance with data reported in literature.^38c^

### Synthesis of CH_2_(O-*p*-C_6_H_4_*t-*Bu)_2_ from *p*-*tert*-Butylphenol and Sodium Hydroxide

Sodium hydroxide (0.37 g, 9.25 mmol) was ground and added to an ampule with an airtight PTFE valve and suspended in 15 mL of dried dichloromethane. Afterward, 1.15 g of *p*-*tert*-butylphenol (7.66 mmol) was added to the mixture, which caused the appearance of a white sticky solid, sodium *p*-*tert*-butylphenoxide. A solution of 15 mg of catalyst **2a** (0.4 mol %) in 5 mL of dried dichloromethane was added with a syringe directly upon the suspension. The resulting colorless mixture was stirred at 60 °C for 24 h in an oil bath preset at the specified temperature. The stirring was quite hindered at the beginning due to the formation of a water phase. After a few minutes at 60 °C, an intense yellow-orange color in the solution. Once the reaction finished, the stirring was stopped. The organic solution was separated from the solid by filtration via cannula. The mixture was filtrated through a pad of silica gel in order to remove the traces of the catalyst. Afterward, the solvent was removed under a vacuum to afford a white powder, in 85% yield (1.02 g). ^1^H NMR (CDCl_3_, 400 MHz): δ 7.31 (m, 4H), 7.04 (m, 4H), 5.69 (s, 2H, C*H*_2_), 1.29 (s, 18H). ^13^C{^1^H} NMR (CDCl_3_, 100 MHz): δ 155.0,145.3,126.5, 116.1, 91.6, 34.3, 31.6. IR (cm^—1^): 3041 (ν C_Ar_—H), 2957 (ν C_sp3_—H), 1509 (ν C_Ar_=C_Ar_), 1211 (ν_as_ C_Ar_—O—C), 1016 (ν_s_ C_Ar_—O—C). Anal. Calcd for C_21_O_28_O_2_: C, 80.73%; H, 9.03%. Found: C, 81.16%; H, 8.87%.

### Synthesis of CH_2_(OCH_2_CH=CH_2_)_2_ from Allyl Alcohol and Sodium Hydroxide

Sodium hydroxide (0.40 g, 9.25 mmol) was ground and added to an ampule with an airtight PTFE valve and suspended in 15 mL of dried dichloromethane. Afterward, 0.48 g of allyl alcohol (8.25 mmol) was added to the mixture, which caused the appearance of a white sticky solid, sodium allyloxide. A solution of 8.25 mg of catalyst **2a** (0.2 mol %) in 5 mL of dried dichloromethane was added with a syringe directly upon the suspension. The resulting colorless mixture was stirred at 60 °C for 24 h in an oil bath preset at the specified temperature. The stirring was quite hindered at the beginning due to the formation of a water phase. After a few minutes at 60 °C, an intense yellow-orange color in the solution was observed. Once the reaction finished, the stirring was stopped. The organic solution was separated from the solid by filtration via cannula. In order to remove the unreacted alcohol, 500 mg of sodium hydroxide was added to the solution, and it was stirred for 1 h. The mixture was filtrated through a pad of silica gel in order to remove the traces of the catalyst. Afterward, the solvent was removed by heating slightly to afford a pale-yellow liquid in 83% yield (0.44 g). ^1^H NMR (CDCl_3_, 400 MHz): δ 5.92 (ddt, *J* = 17.2 Hz, *J* = 10.4, *J* = 5.6 Hz), 5.29 (m, *J* = 17.2 Hz, *J* = 1.6 Hz, 2H), 5.18 (m, *J* = 10.4 Hz, *J* = 1.6 Hz, 2H), 4.72 (s, 2H), 4.08 (m, *J* = 5.6 Hz, *J* = 1.5 Hz, 4H). ^13^C{^1^H} NMR (CDCl_3_, 100 MHz): δ 134.5,117.2, 93.9, 68.5. IR (cm^—1^): 3081 (ν C_sp2_—H), 2881 (ν C_sp3_—H), 1647 (ν C=C), 1105 (ν_as_ C—O—C), 1041 (ν_s_ C—O—C), 918 (δ C=CH_2_). Spectroscopic data is in accordance with data reported in literature.^38c^

### Synthesis of CH_2_(O-*i*-Pr)_2_ from 2-Propanol and Sodium Hydride

Sodium hydride (0.40 g, 9.25 mmol) was added to an ampule with an airtight PTFE valve and washed with hexane in order to remove the oil suspension. The dried solid was suspended in 15 mL of dichloromethane. Afterward, 0.50 g of 2-propanol (8.25 mmol) was added to the mixture, which caused the appearance of a white sticky solid, sodium 2-propoxide. A solution of 8.25 mg of catalyst **2a** (0.2 mol %) in 5 mL of dried dichloromethane was added with a syringe directly upon the suspension. The resulting colorless mixture was stirred at 60 °C for 24 h in an oil bath preset at the specified temperature. After a few minutes at 60 °C, an intense yellow-orange color in the solution was observed. Once the reaction finished, the stirring was stopped. The organic solution was separated from the solid by filtration via cannula. In order to remove the unreacted alcohol, 500 mg of sodium hydroxide was added to the solution, and it was stirred for 1 h. The mixture was filtrated through a pad of silica gel in order to remove the traces of the catalyst. Afterward, the solvent was removed by heating slightly to afford a pale-yellow liquid in 67% yield (0.36 g). ^1^H NMR (CDCl_3_, 400 MHz): δ 4.72 (s, 2H), 3.90 (sep, *J* = 6.2, 2H), 1.17 (d, *J* = 6.2, 12H). ^13^C{^1^H} NMR (CDCl_3_, 100 MHz): δ 90.9,68.6, 22.6. IR (cm^–1^): 2921 (ν C_sp3_—H), 1033 (ν_s_ C—O—C). Spectroscopic data is in accordance with data reported in literature.^38e^

### Synthesis of CH_2_(SEt)_2_ from Ethanethiol and Sodium Hydroxide

Sodium hydroxide (0.55 g, 13.75 mmol) was ground and added to an ampule with an airtight PTFE valve and suspended in 15 mL of dichloromethane. Afterward, 0.80 g of ethanethiol (12.9 mmol) was added to the mixture, which caused the appearance of a white sticky solid, sodium ethanethiolate. A solution of 25 mg of catalyst **2a** (0.4 mol %) in 5 mL of dichloromethane was added with a syringe directly upon the suspension. The resulting colorless mixture was stirred at 60 °C for 24 h in an oil bath preset at the specified temperature. The stirring was quite hindered at the beginning due to the formation of a water phase. Once the reaction finished, the stirring was stopped. The organic solution was separated from the solid by filtration via cannula. No further purification was performed. Conversion >99% by NMR was found. ^1^H NMR (CDCl_3_, 400 MHz): δ 3.69 (s, 2H), 2.65 (q, *J* = 7.4 Hz, 4H), 1.26 (t, *J* = 7.4 Hz, 6H). Spectroscopic data is in accordance with data reported in literature.^38f^

### Synthesis of CH_2_Pz_2_ from Pyrazole and Sodium Hydride

Sodium hydride (0.60 g, 24.9 mmol) was added to an ampule with an airtight PTFE valve and washed with hexane in order to remove the oil suspension. The dried solid was suspended in 15 mL of dichloromethane. Afterward, 1.13 g (16.6 mmol) of pyrazole was added to the suspension. The addition led to the formation of sodium pyrazolate and hydrogen. A solution of 16.5 mg of catalyst **2a** (0.2 mol %) in 5 mL of dichloromethane was added with a syringe directly upon the suspension, and it is stirred at 60 °C for 24 h in an oil bath preset at the specified temperature. After a few minutes at 60 °C, a brownish white color in the solution was observed. Once the reaction finished, the stirring was stopped. The organic solution was separated from the solid by filtration via cannula. The solvent was removed under a vacuum to afford a pale-brown solid. The solid is purified by crystallization in heptane, where the product is soluble at high temperatures, and the impurities remain insoluble, to afford a white solid, in 72% yield (0.88 g). ^1^H NMR (CDCl_3_, 400 MHz): δ 7.65 (dd, *J* = 2.4 Hz, *J* = 0.6 Hz, 2H), 7.55 (dd, *J* = 1.9 Hz, *J* = 0.6 Hz, 2H), 6.31 (s, 2H), 6.29 (dd, *J* = 2.4 Hz, *J* = 1.9 Hz, 2H). ^13^C{^1^H} NMR (CDCl_3_, 100 MHz): δ 141.0, 129.8, 107.3, 65.5. IR (cm^—1^): 3101 (ν C_Ar_–H), 2924 (ν C_sp3_—H), 1610 (ν C_Ar_=N), 1514 (ν C_Ar_=C_Ar_), 1435 (ν C_Ar_=C_Ar_). Spectroscopic data is in accordance with data reported in literature.^38g^

### Synthesis of [MeOCH_2_·**1a**]^+^[Br^–^] (**3a**)

To a 50 mL Schlenk flask was added 100 mg (0.22 mmol) of **1a**. The yellow solid was dissolved in 10 mL of dried dichloromethane to afford a clear yellow solution. Then, 20 μL (0.22 mmol, 90%) of bromomethyl methyl ether was added, and the solution was stirred for 30 min while the color faded. Afterward, 10 mL of dried hexane was added to the solution, and the solvent was removed under a vacuum. The solid was washed with more hexane, to afford yellowish solid with a yield of 94% (0.12 g). ^1^H NMR (CD_2_Cl_2_, 400 MHz): δ 8.30 (s, 2H), 7.15 (m, 2H), 7.04 (m, 4H), 6.43 (m, 2H), 5.25 (br s, 2H), 4.06 (m, 2H), 3.47 (s, 3H), 2.30 (s, 3H), 2.25 (s, 3H), 1.90 (m, 2H), 1.79 (m, 4H), 1.67 (m, 4H), 1.50 (m, 2H), 1.34 (m, 4H), 1.23 (m, 2H), 0.99 (m, 2H). ^13^C{^1^H} NMR (CD_2_Cl_2_, 100 MHz): δ 143.3, 138.4, 136.1, 134.1, 130.8, 130.7, 124.7, 123.1, 121.0, 82.6, 60.4, 57.5, 33.5, 33.3, 25.9, 25.8, 24.7, 21.0, 20.9. Elemental analysis calcd for C_32_H_43_ON_4_Br: C, 66.31; H, 7.48; N, 9.67. Found: C, 66.46; H, 7.22; N, 9.43. ESI-MS (1 μM in anhydrous THF): *m*/*z* 499.56 (MeOCH_2_·**1a**^+^). HRMS (ESI) *m*/*z*: [M]^+^ calcd for C_32_H_43_N_4_O, 499.3431; found, 499.3423.

### Spectroscopic Data of **1b**

In order to detect and characterize **1b**, the general procedure of catalysis was applied to obtain methylal starting from NaOMe. Once the reaction showed an intense change of color to a deep orange-yellow, the mixture was filtered, and the liquid phase was dried under a vacuum. An oily deep red solid was obtained. No further purification was performed. ^1^H NMR (CD_2_Cl_2_, 400 MHz): δ 6.98 (m, 2H). 6.93 (m, 2H), 6.90 (m, 2H), 6.65 (m, 2H), 4.69 (s, 2H), 3.75 (s, 3H), 3.30 (s, 3H), 2.24 (s, 3H), 2.20 (s, 3H). ^13^C{^1^H} NMR (CD_2_Cl_2_, 100 MHz): δ 152.5, 145.2, 141.8, 134.3, 131.4, 129.5, 123.5, 121.9, 82.4, 55.7,55.7, 20.8, 20.8. ESI-MS (1 μM THF/wet methanol 1:100): *m*/*z* 299.30 (**1b**·H^+^, 100%); 321.30 (**1b**·Na^+^, 73%); 337.33 (**1b**·K^+^, 13%). HRMS (ESI) *m*/*z*: [M + H]^+^ calcd for C_18_H_23_N_2_O_2_, 299.1754; found, 299.1753. HRMS (ESI) *m*/*z*: [M + Na]^+^ calcd for C_18_H_22_N_2_O_2_Na, 321.1573; found, 321.1770.

### Spectroscopic Data of **2b**

In order to detect and characterize **2b**, the general procedure of catalysis was applied to obtain methylal starting from NaOMe. Once the reaction showed an intense change of color to a deep orange-yellow, the mixture was filtered, and the liquid phase was dried under a vacuum. An oily, deep red solid was obtained. No further purification was performed. ^1^H NMR (CD_2_Cl_2_, 400 MHz): δ 6.97 (m, 4H), 6.88 (m, 4H), 6.86 (m, 4H), 6.54 (m, 4H), 5.15 (s, 2H), 3.53 (s, 6H), 2.25 (s, 6H), 2.20 (s, 6H). ^13^C{^1^H} NMR (CD_2_Cl_2_, 100 MHz): δ 152.5, 145.5, 141.1, 135.2, 131.2, 129.5, 129.4, 125.9, 122.2, 67.1, 55.9, 20.9, 20.8. ESI-MS (1 μM in THF/wet methanol 1:100): *m*/*z* 521.51 (**2b**·H^+^, 100%); 543.50 (**2b**·Na^+^, 19%); 559.45 (**2b**·K^**+**^, 7%). HRMS (ESI) *m*/*z*: [M + H]^+^ calcd for C_33_H_37_N_4_O_2_, 521.2911; found, 521.2902. HRMS (ESI) *m*/*z*: [M + Na]^+^ calcd for C_33_H_36_N_4_O_2_Na, 343.2730; found, 343.2719.

## Supporting Information Available

Additional experimental procedures and spectroscopic data (PDF)

## Abbreviations

NHC: N-heterocyclic carbene
CDI: carbodiimide
DCM: dichloromethane
ICy: 1,3-dicyclohexylimidazolylidene
PTBP: *p*-*tert*-butylphenol
AA: allyl alcohol
HPz: pyrazole
TON: turnover number
TOF: turnover frequency
NMR: nuclear magnetic resonance
DOSY: diffusion-ordered spectroscopy
HSQC: heteronuclear single quantum coherence spectroscopy
HMBC: heteronuclear multiple bond correlation
