# Supplementary material for: NHC-CDI Betaine Adducts and Their Cationic Derivatives as Catalyst Precursors for Dichloromethane Valorization
Source: J Org Chem. 2021 Nov 1;86(23):16725–35. doi: 10.1021/acs.joc.1c01971 (PMC8650018; doi:10.1021/acs.joc.1c01971)
Supplement: Supplementary file 1 — jo1c01971_si_001.pdf [file jo1c01971_si_001.pdf]

# Supporting Information

# NHC-CDI Betaine Adducts and their Cationic Derivatives as Catalyst Precursors for the Dichloromethane Valorization

David Sánchez-Roa,[a] Marta E. G. Mosquera*,[a] and Juan Cámpora*,[b]

a) Departamento de Química Orgánica y Química Inorgánica, Instituto de Investigación en Química "Andrés M. del Río" (IQAR) Universidad de Alcalá, Campus Universitario, 28871-Alcala de Henares, Madrid, Spain

b) Instituto de Investigaciones Químicas, CSIC-Universidad de Sevilla, C/ Américo Vespucio, 49, 41092, Sevilla, Spain

*Corresponding authors: martaeg.mosquera@uah.es, campora@iiq.csic.es

## CONTENTS

1. **Experimental methods............S1**
2. **Optimization of the general reaction conditions for the syntheses of methylal from DCM and NaOMe with catalyst 2a............S1**
3. **Spectroscopic data............S2**
4. **References............S16**

## 1. Experimental methods

All manipulations were performed under inert atmosphere using Schlenk-line techniques ($O_2$ < 3 ppm) and a glove box ($O_2$ < 0.6 ppm) MBraun MB-20G. Solvents were purified using an MBraun Solvent Purification System, except from dichloromethane (DCM) and THF, which were distilled with $CaH_2$ and Na respectively. All NMR scale experiments were carried out in sample tubes with air-tight PTFE valves. Deuterated solvents were degassed and stored in the glove box in the presence of molecular sieves (4 Å). NMR spectra were recorded with a Bruker 400 Ultrashield ($^{1}$H 400 MHz, $^{13}$C 101 MHz) at 25 °C. All chemical shifts were determined using residual signals of solvents and were referenced with regard to external $SiMe_4$. Assignments of spectral signals was helped with 2D ($^{1}$H-$^{13}$C HSQC and HMBC) and diffusion (DOSY) NMR experiments. Elemental analysis and ESI-MS spectra of samples were carried out by the Analytical Services of the Institute for Chemical Research (Seville, Spain) using an LECO CHNS-TruSpec and Bruker Ion Trap Bruker Esquire 6000, respectively. HR-ESI spectra were carried out by the Mass Spectrometry Service (CITIUS, University of Seville) in a Thermo Scientific Orbitrap Elite hybrid mass spectrometer, operating with direct injection and ESI ion source and ion-trap analyzer. Unless otherwise specified, all commercial reagents were purchased and from Sigma-Aldrich and used as received. Imidazolium salt[1] **ICy·HBF$_4$** and catalysts[2,3] **1a** and **2a** were prepared according to literature and our synthetic procedures, previously reported. **WARNING:** although dichloromethane does not react vigorously with NaH, NaOH or sodium alkoxides or aryloxides described in this work, strongly basic reagents like potassium tert-butoxide or neat sodium, which are known to react violently with this solvent, should be avoided.

## 2. Optimization of the general reaction conditions for the syntheses of methylal from DCM and NaOMe with catalyst 2a

**Table S1. Reaction conditions and conversion for methylal syntheses from NaOMe and DCM using 2a under different conditions[a]**

| $V_{DCM}$ (mL, mmol) | NaOMe (g, mmol) | 2a (mg, mol %) | temp. (°C), t (h) | Methylal/DCM | NMR yield (%) |
|---|---|---|---|---|---|
| 5 mL (78 mmol) | 108 mg (2 mmol) | 20 mg (2 mol%) | 25 °C, 24 h | 1/77 | 97 % |
| 5 mL (78 mmol) | 108 mg (2 mmol) | 10 mg (1 mol%) | 25 °C, 24 h | 1/77 | 98 % |
| 10 mL (156 mmol) | 1.08 g (20 mmol) | 10 mg (0.1 mol%) | 25 °C, 24 h | 10/146 | 97 % |
| 20 mL (313 mmol) | 2.00 g (37 mmol) | 184 mg ( 1 mol %) | 25 °C, 24 h | 18/295 | 99% |
| 20 mL (313 mmol) | 2.00 g (37 mmol) | 37 mg (0.2 mol%) | 60 °C, 24 h | 18/295 | 99 % |
| 40 mL (626 mmol) | 20.0 g (0.37 mol) | 18 mg (0.01 mol%) | 60 °C, 24 h | 185/441 | 99% |

(a) (all % figures based on NaOMe)

## 3. Spectroscopic data

### $CH_2(OMe)_2$

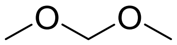

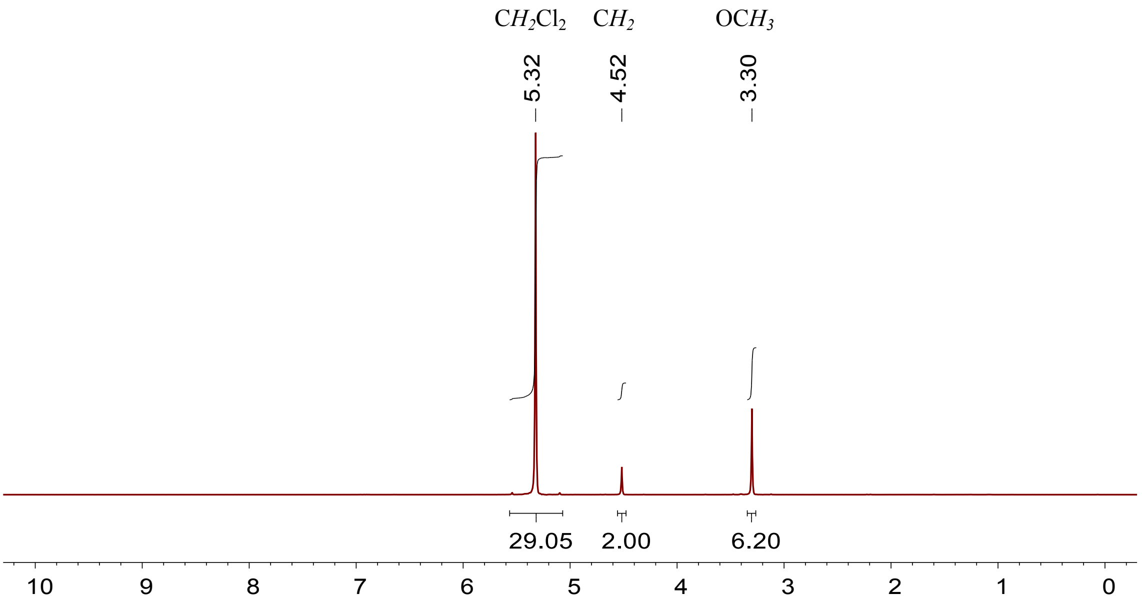


**Figure S1. $^1$H-NMR (400 MHz) of $CH_2(OMe)_2$ in $CDCl_3$. Peak at 5.32 belongs to DCM from the reaction**

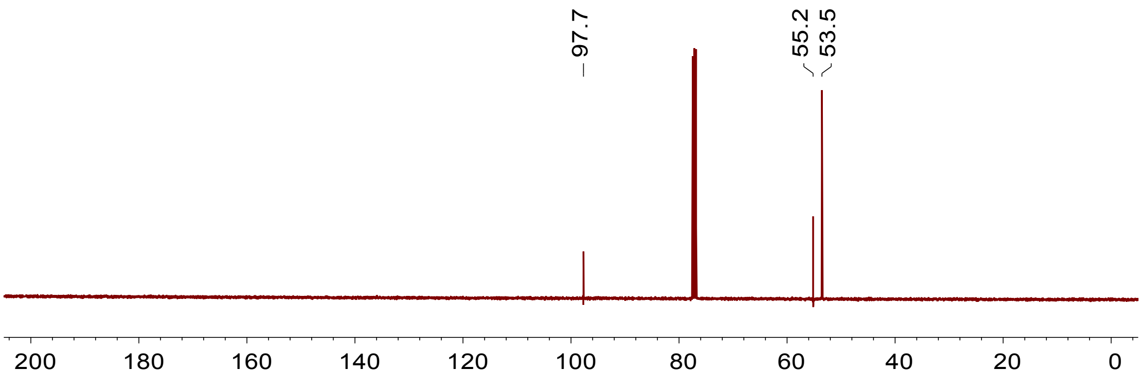


**Figure S2. $^{13}C\{^1H\}$-NMR (101 MHz) of $CH_2(OMe)_2$ in $CDCl_3$. Peak at 53.5 belongs to DCM from the reaction**

**$CH_2(OPh)_2$**

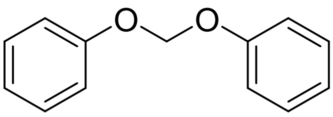


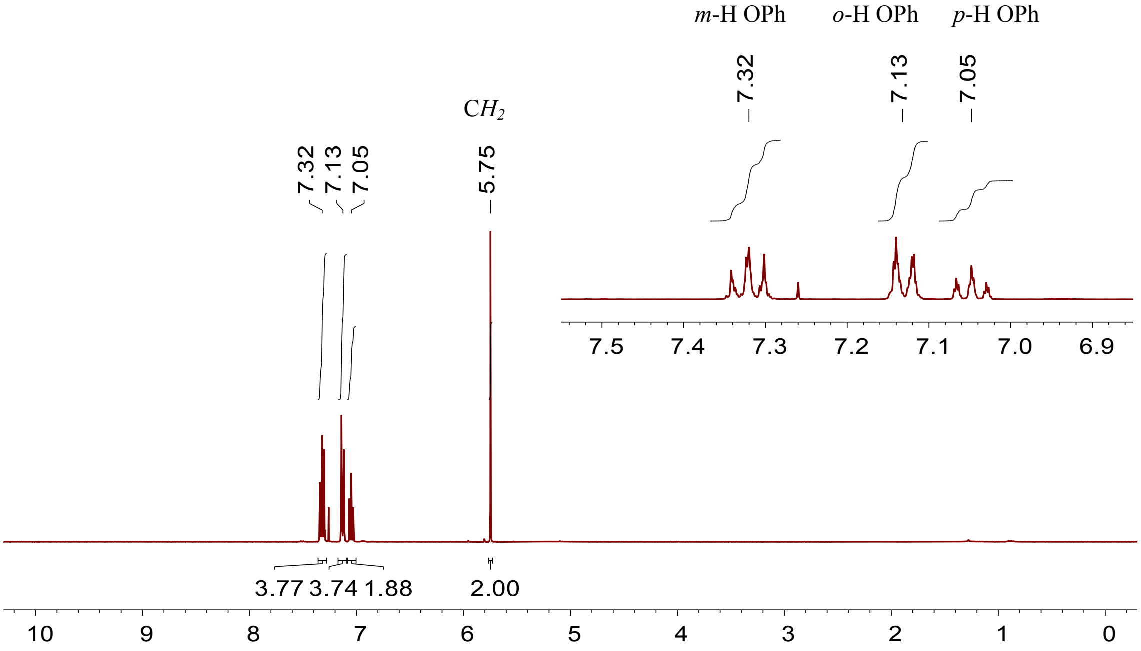


**Figure S3.** **$^{1}$H-NMR (400 MHz) of $CH_2(OPh)_2$ in $CDCl_3$**

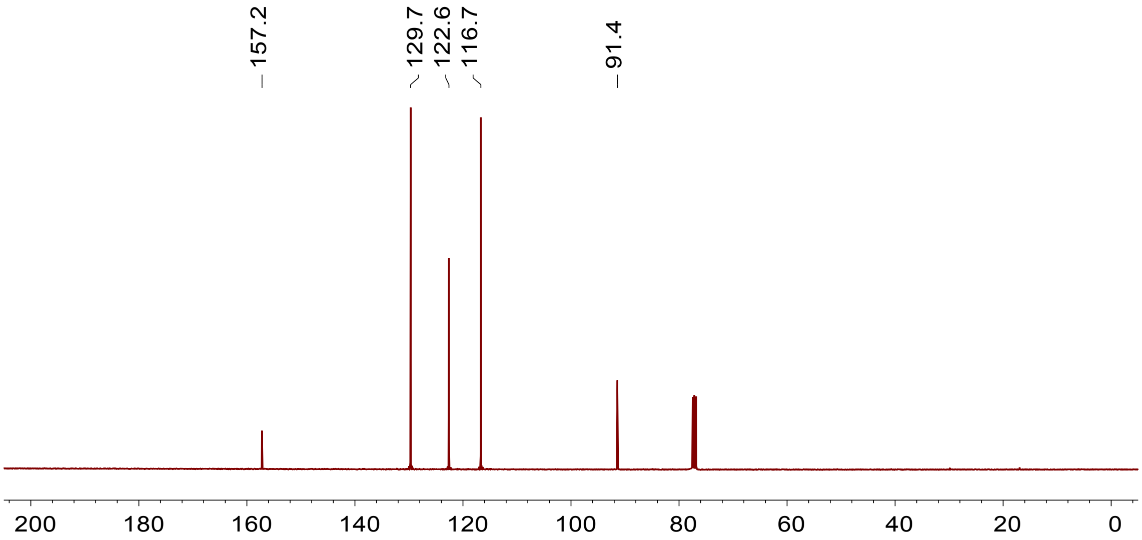


**Figure S4.** **$^{13}C\{^{1}H\}$-NMR (101 MHz) of $CH_2(OPh)_2$ in $CDCl_3$**

**$CH_2(OBn)_2$**

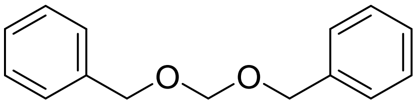


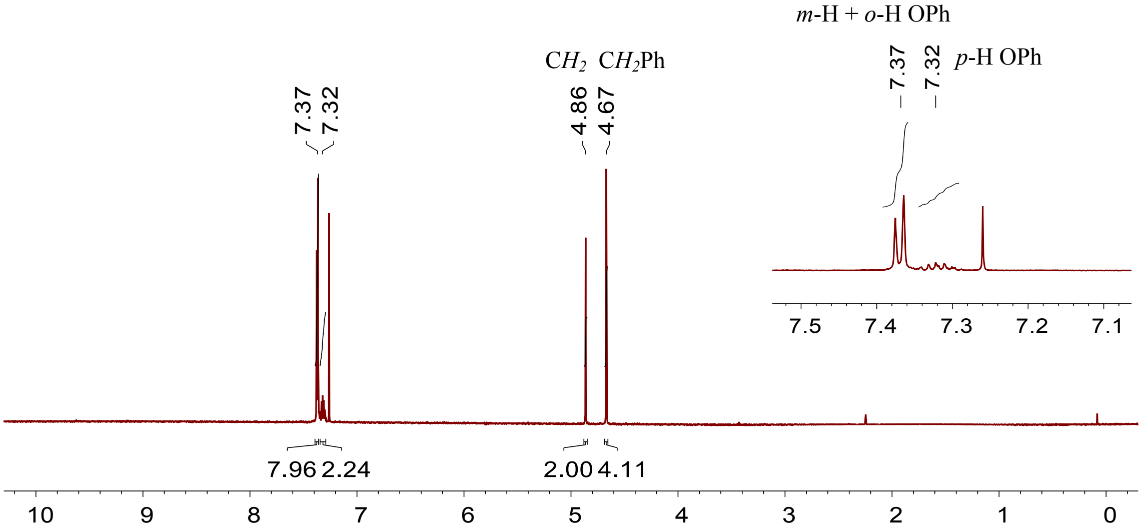


**Figure S5. $^1$H-NMR (400 MHz) of $CH_2(OBn)_2$ in $CDCl_3$**

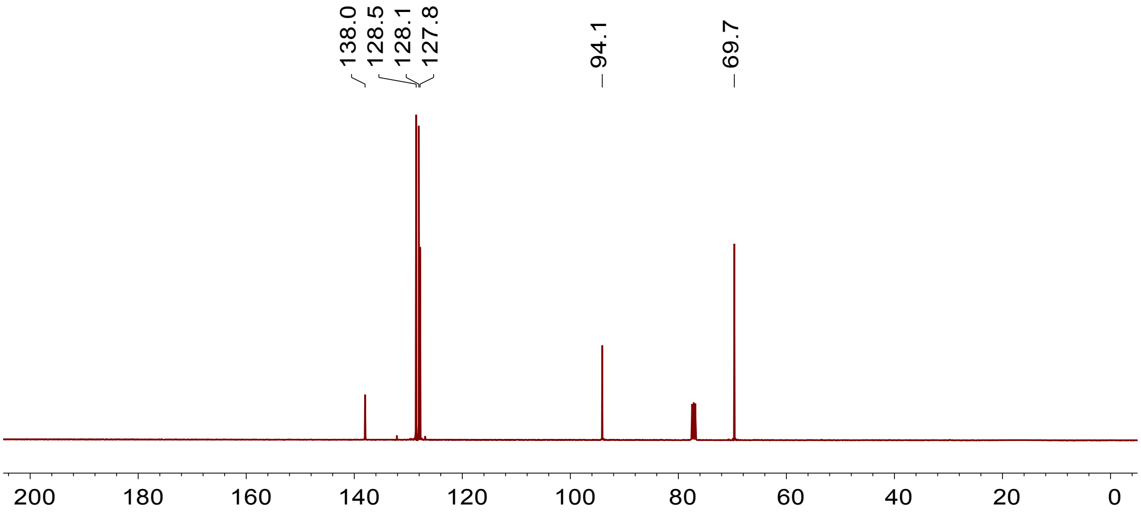


**Figure S6. $^{13}C\{^1H\}$-NMR (101 MHz) of $CH_2(OBn)_2$ in $CDCl_3$**

**$CH_2$(OPh-*p*-*t*-Bu)$_2$**

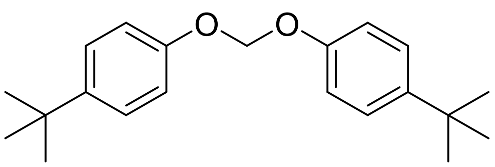


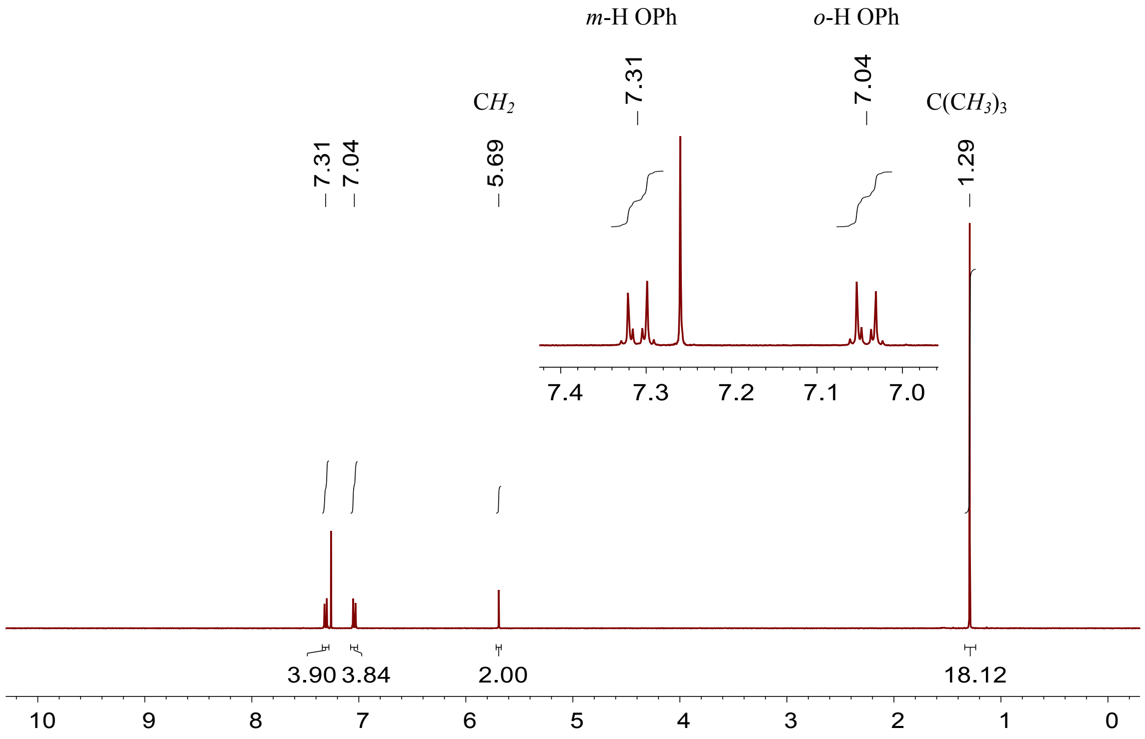


**Figure S7. $^{1}$H-NMR (400 MHz) of $CH_2$(OPh-*p*-*t*-Bu)$_2$ in $CDCl_3$**

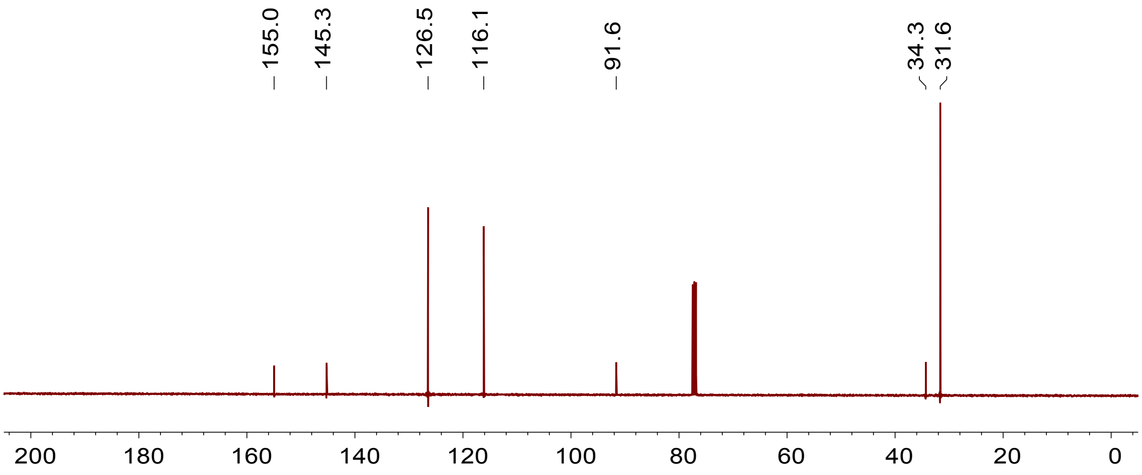


**Figure S8. $^{13}$C{$^{1}$H}-NMR (101 MHz) of $CH_2$(OPh-*p*-*t*-Bu)$_2$ in $CDCl_3$**

**$CH_2(OCH_2CH{=}CH_2)_2$**

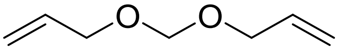


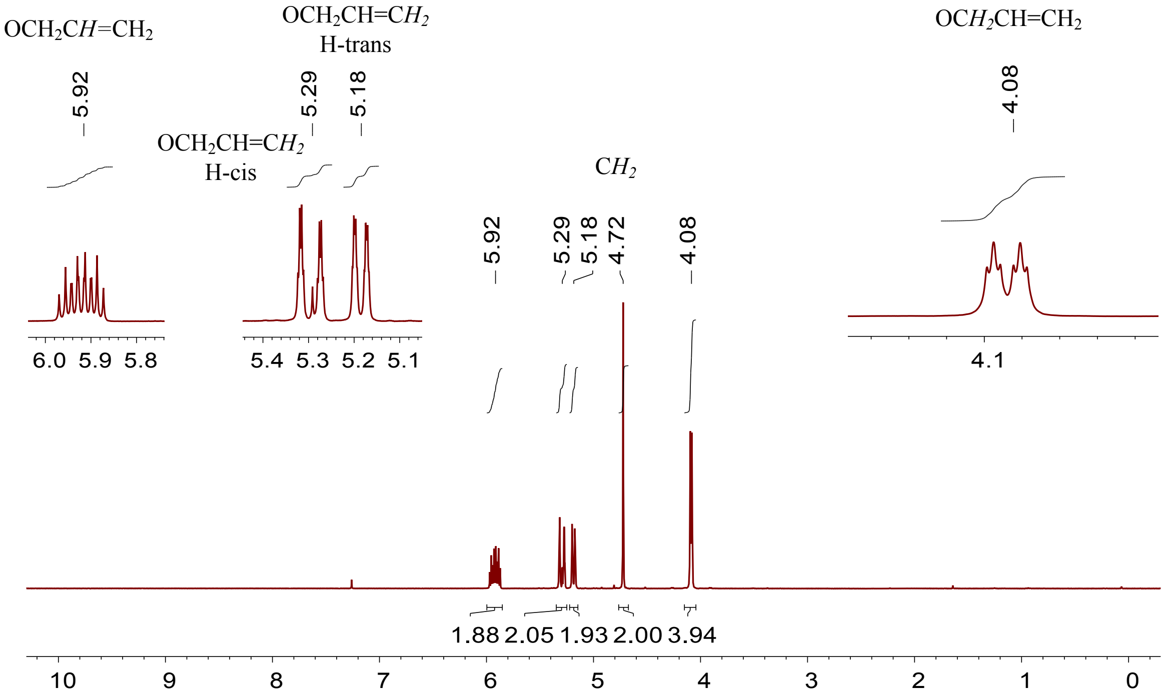


**Figure S9. $^1$H-NMR (400 MHz) of $CH_2(OCH_2CH{=}CH_2)_2$ in $CDCl_3$**

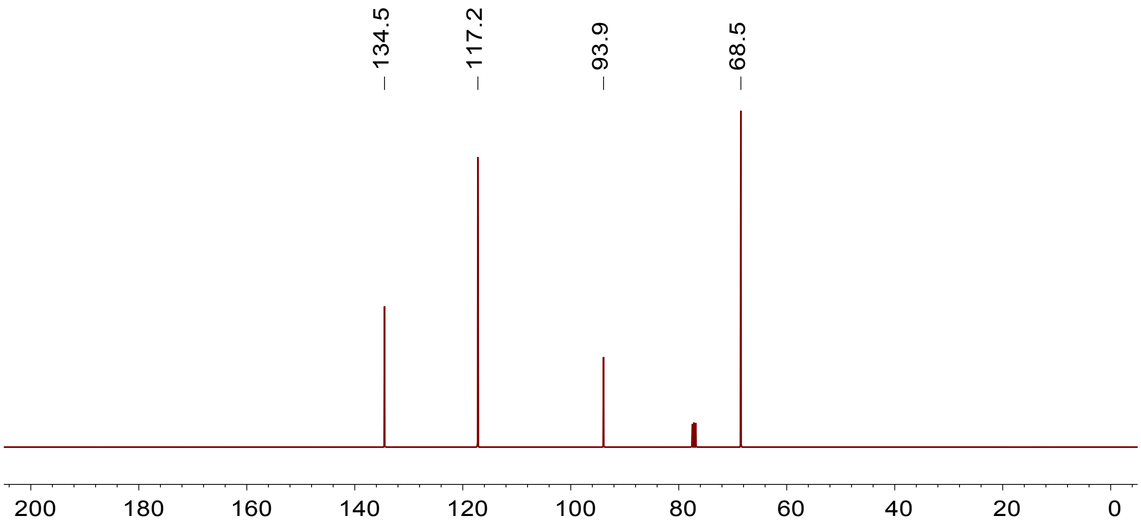


**Figure S10. $^{13}C\{^1H\}$-NMR (101 MHz) of $CH_2(OCH_2CH{=}CH_2)_2$ in $CDCl_3$**

**$CH_2(O\text{-}i\text{-}Pr)_2$**

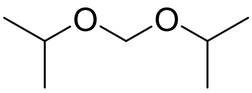

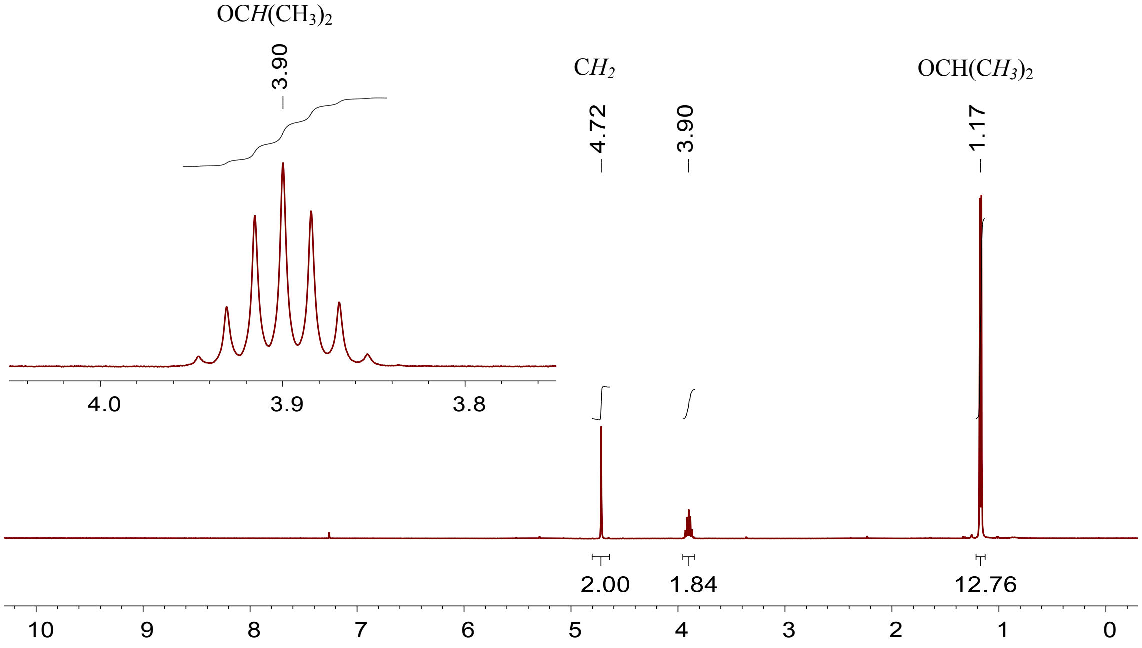


**Figure S11.** **$^1$H-NMR (400 MHz) of $CH_2(O\text{-}i\text{-}Pr)_2$ in $CDCl_3$**

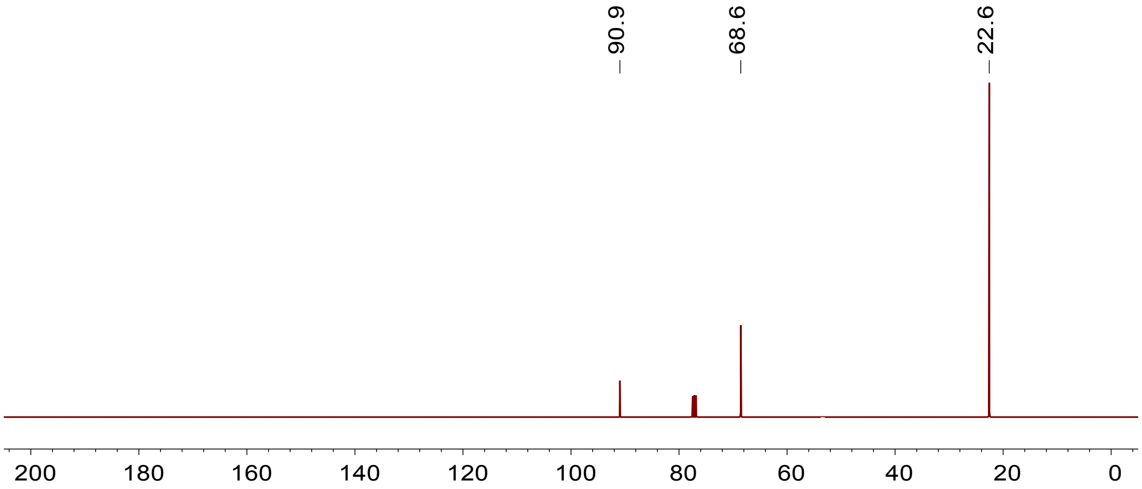


**Figure S12.** **$^{13}C\{^1H\}$-NMR (101 MHz) of $CH_2(O\text{-}i\text{-}Pr)_2$ in $CDCl_3$**

**$CH_2(SEt)_2$**

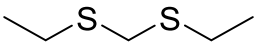

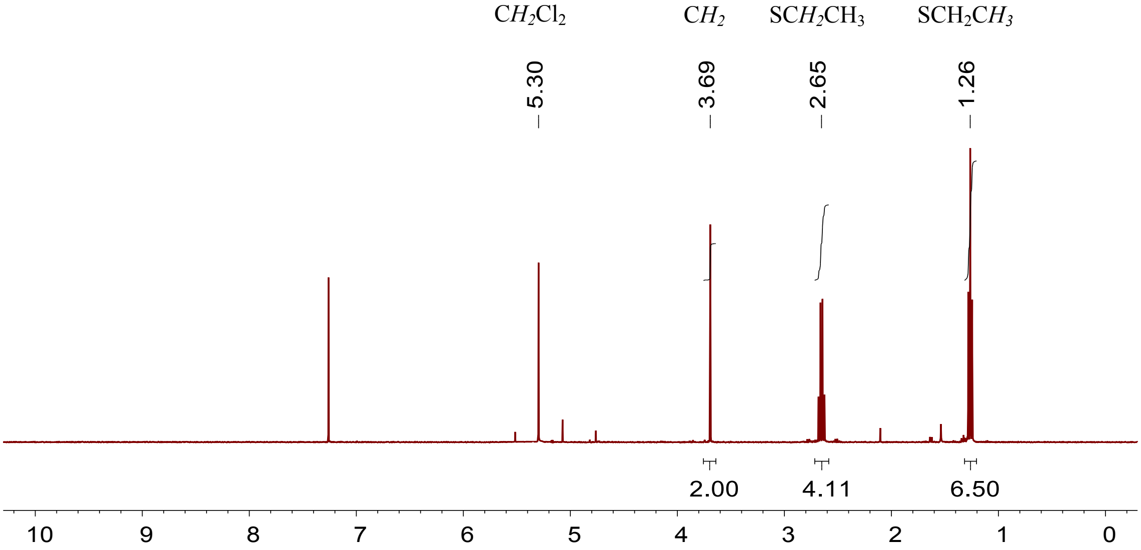


**Figure S13.** **$^{1}$H-NMR (400 MHz) of $CH_2(SEt)_2$ in $CDCl_3$. Peak at 5.30 belongs to DCM from the reaction**

**$CH_2Pz_2$**

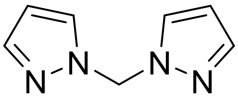

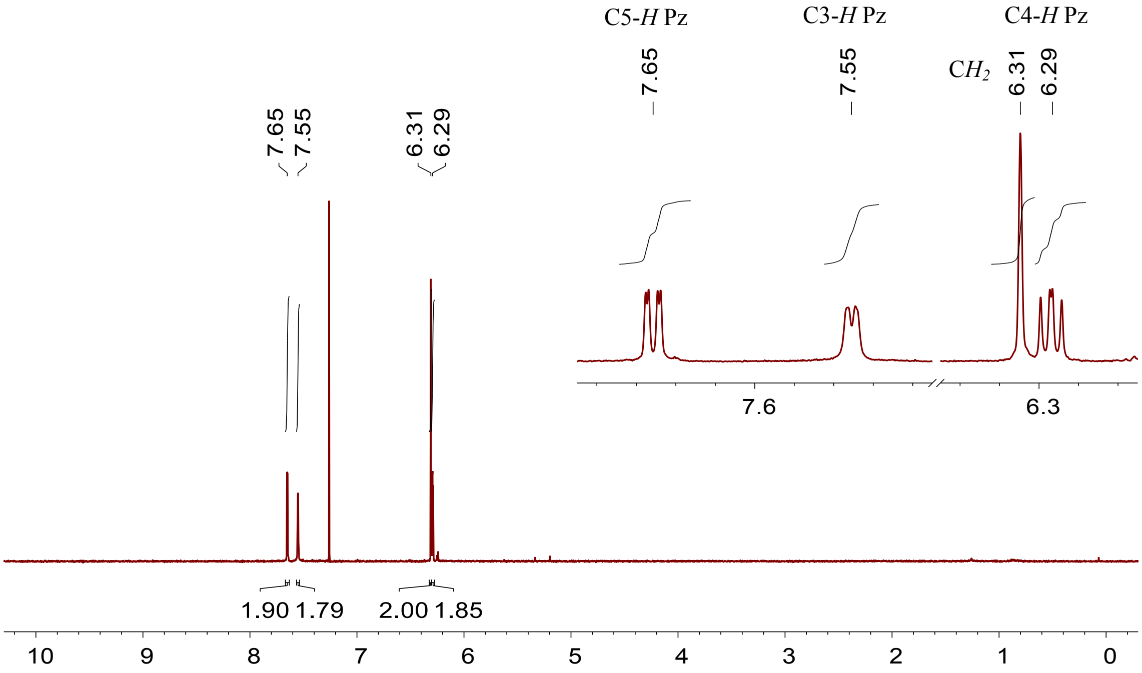


**Figure S14.** **$^1$H-NMR (400 MHz) of $CH_2Pz_2$ in $CDCl_3$**

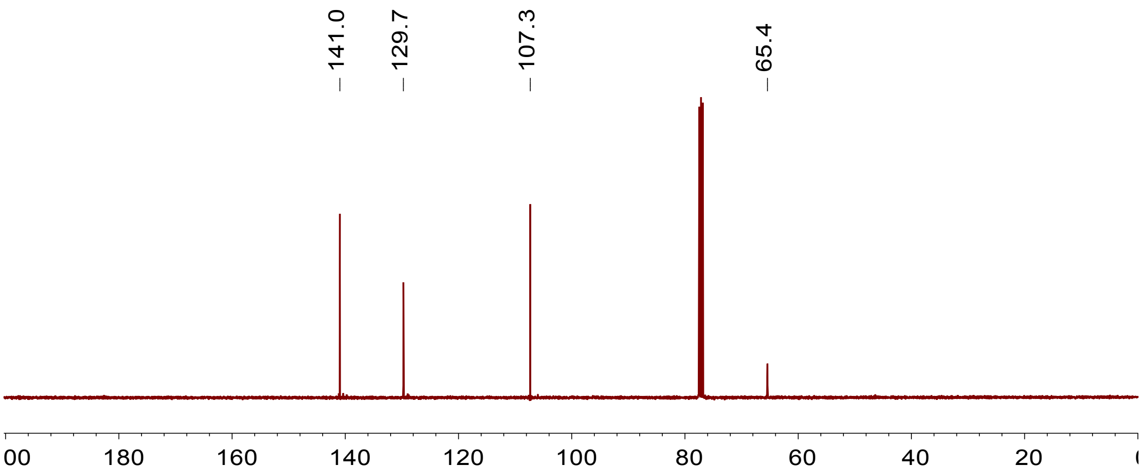


**Figure S15.** **$^{13}C\{^1H\}$-NMR (101 MHz) of $CH_2Pz_2$ in $CDCl_3$**

**1b**

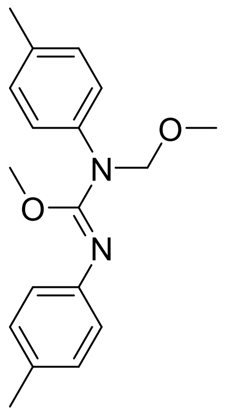


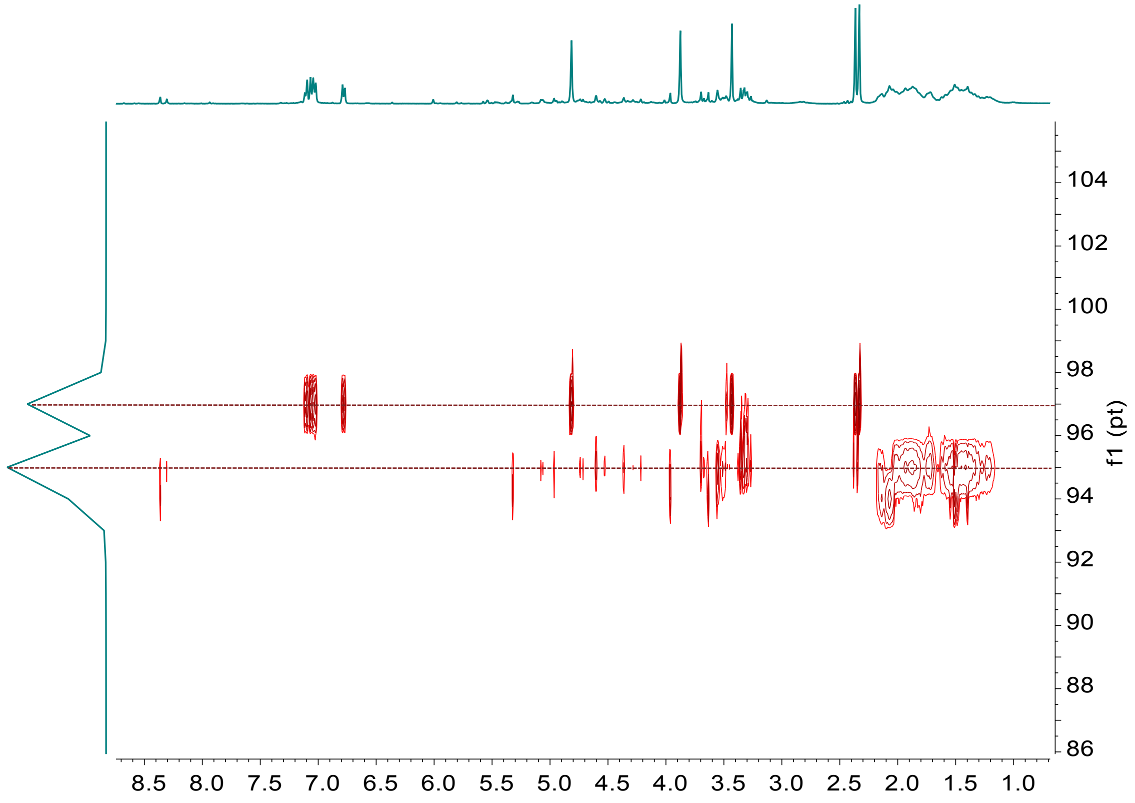


**Figure S16. DOSY spectrum (400 MHz) of 1b (catalytic medium) in $CD_2Cl_2$**

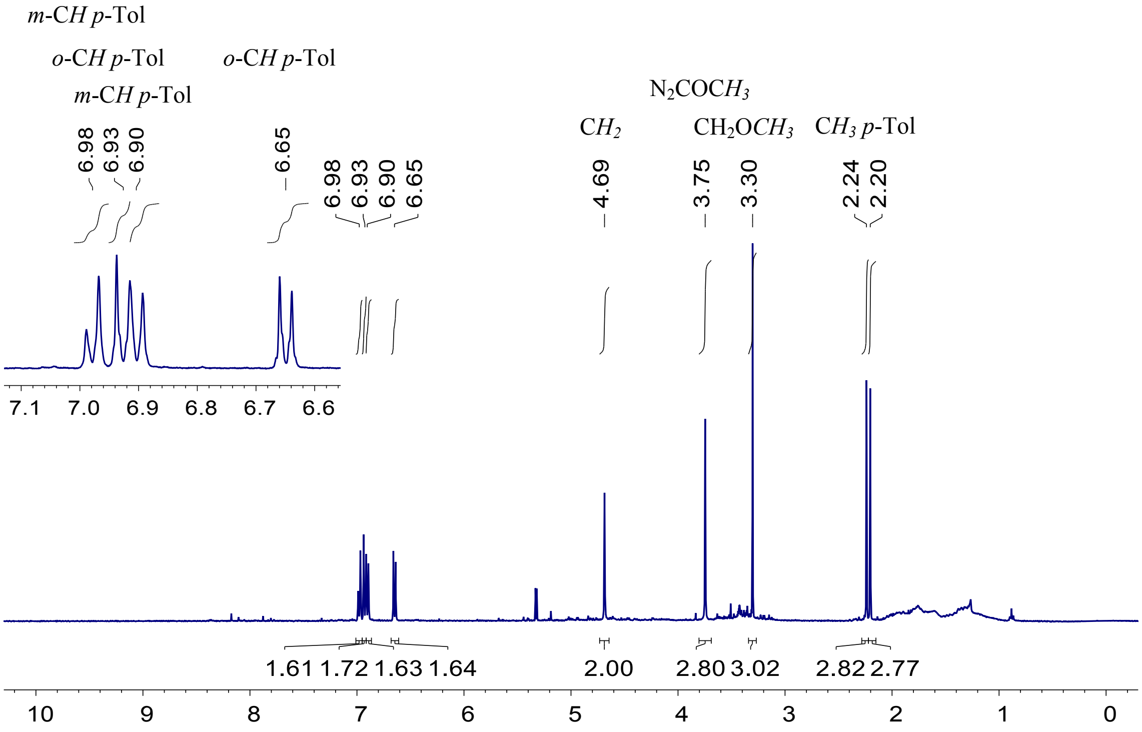


**Figure S17.** **$^{1}$H-NMR (400 MHz) of 1b (catalytic medium) in $CD_2Cl_2$**

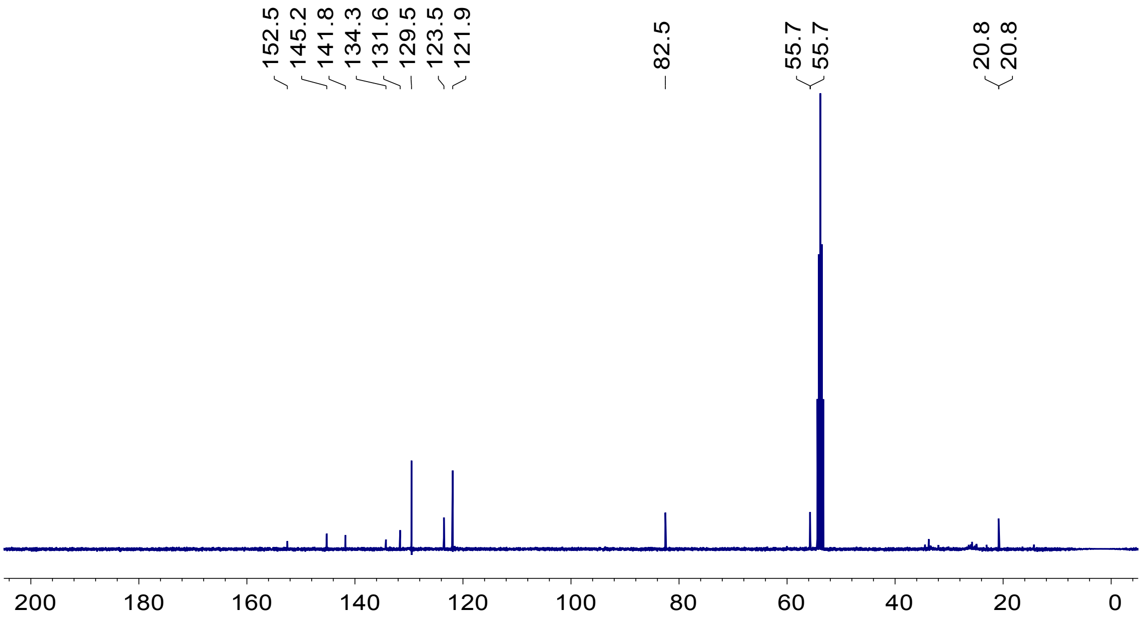


**Figure S18.** **$^{13}$C{$^{1}$H}-NMR (101 MHz) of 1b (catalytic medium) in $CD_2Cl_2$**

**2b**

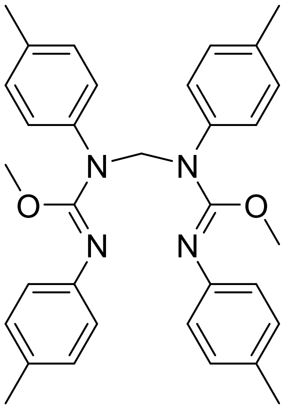


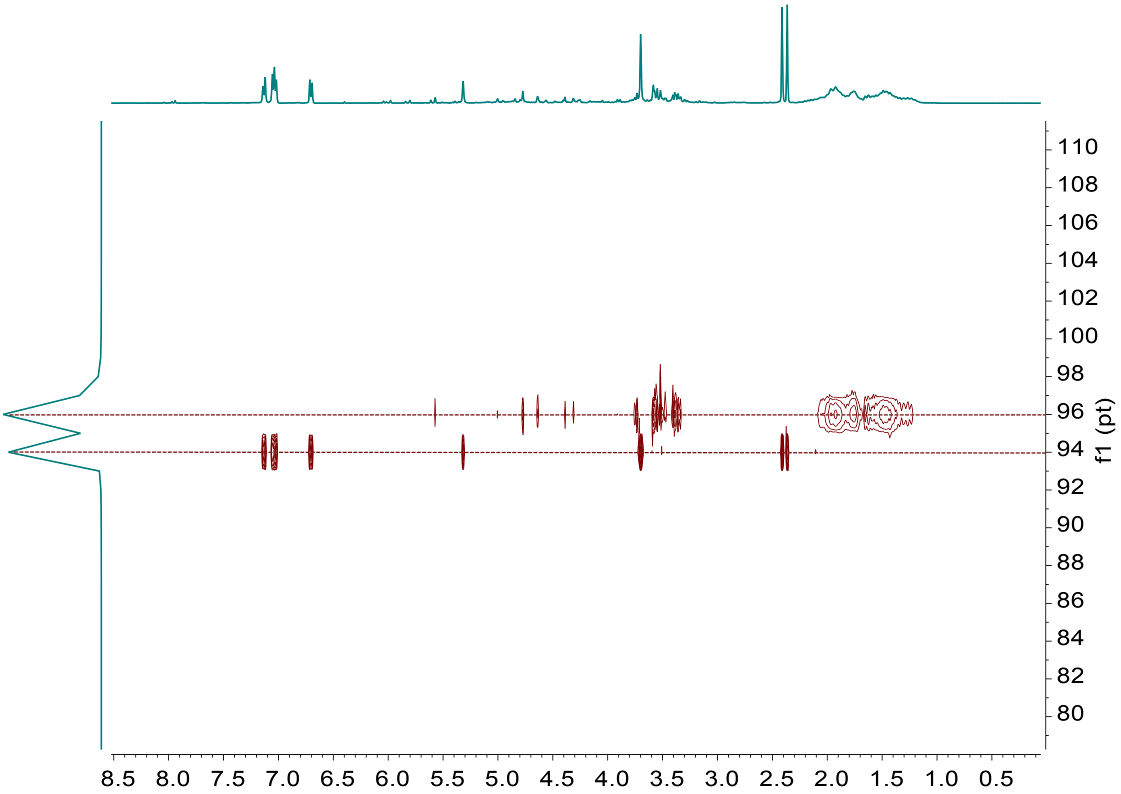


**Figure S19. DOSY spectrum (400 MHz) of 2b (catalytic medium) in $CD_2Cl_2$**

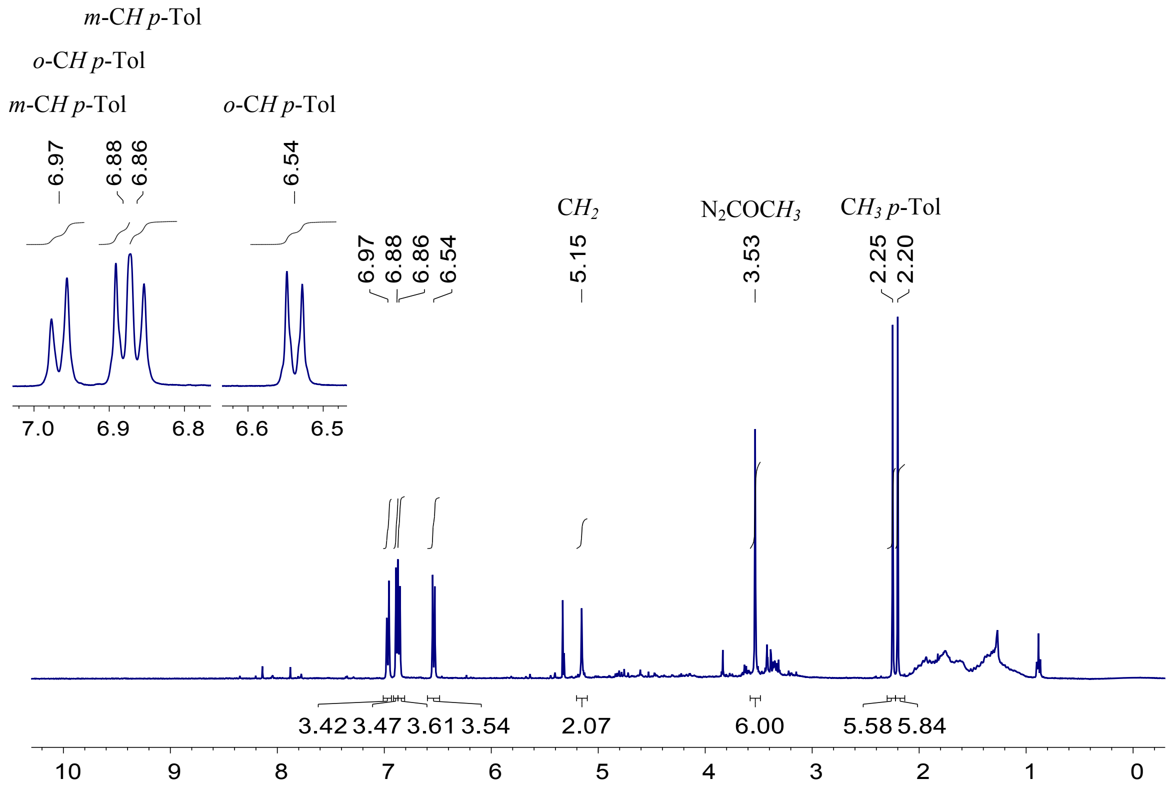


**Figure S20.** **$^{1}$H-NMR (400 MHz) of 2b (catalytic medium) in $CD_2Cl_2$**

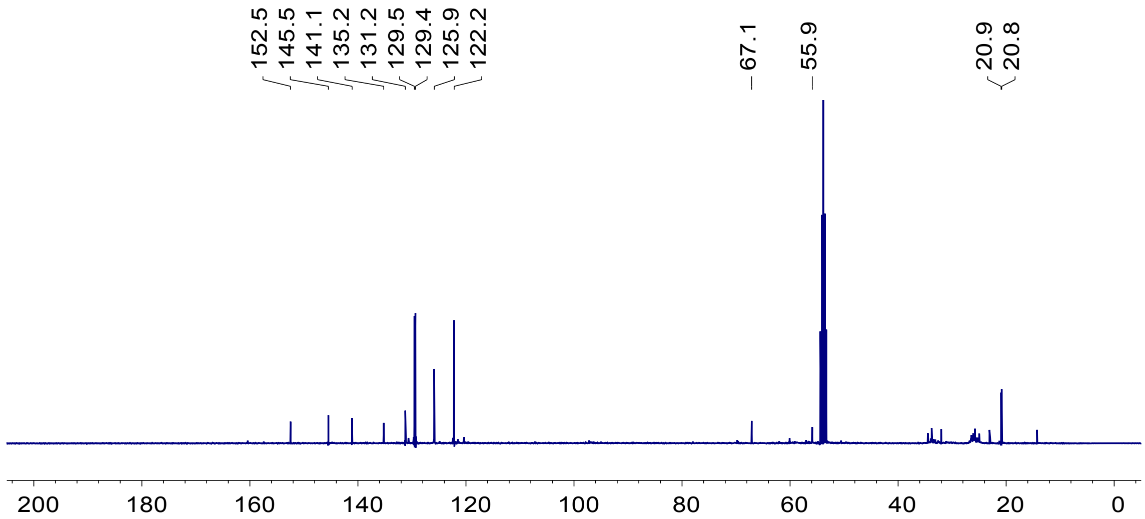


**Figure S21.** **$^{13}C\{^{1}H\}$-NMR (101 MHz) of 2b (catalytic medium) in $CD_2Cl_2$**

**[MeOCH$_2$·1a]$^+$[Br$^-$] (3a)**

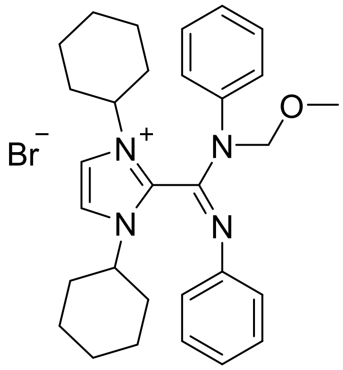


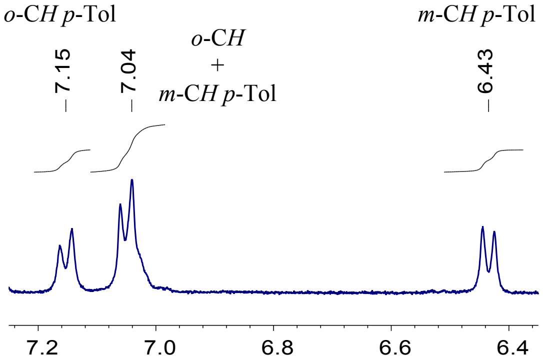


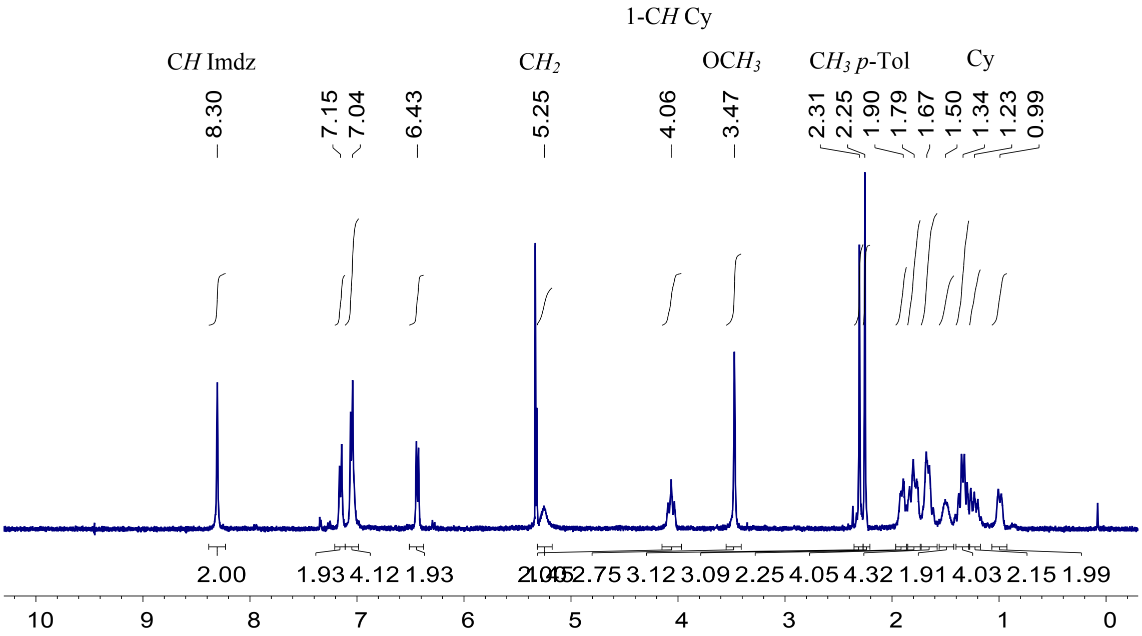


**Figure S22.** $^1$**H-NMR (400 MHz) of 3a in CD$_2$Cl$_2$**

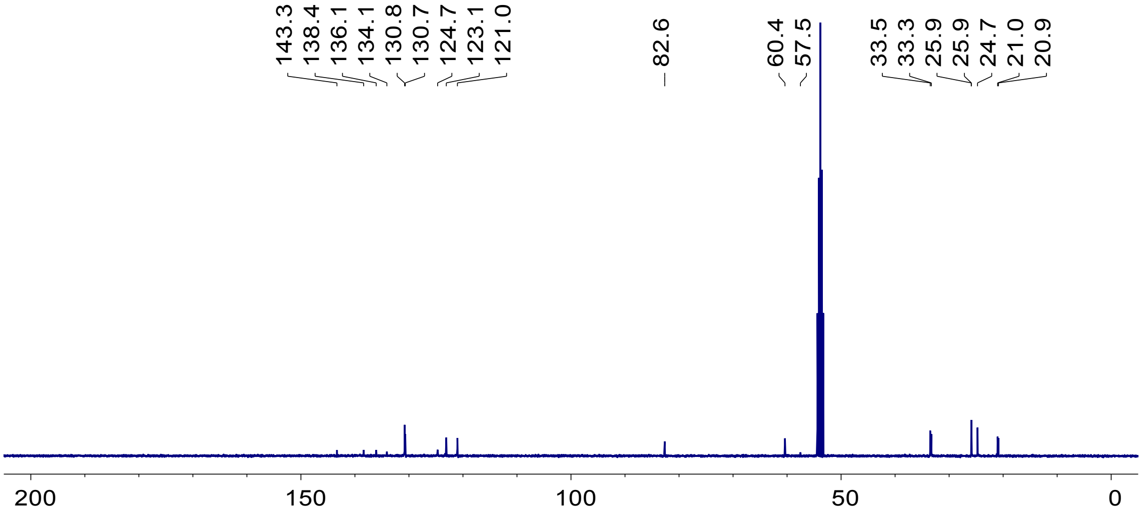


**Figure S23.** $^{13}$**C{$^1$H}-NMR (101 MHz) of 3a in CD$_2$Cl$_2$**

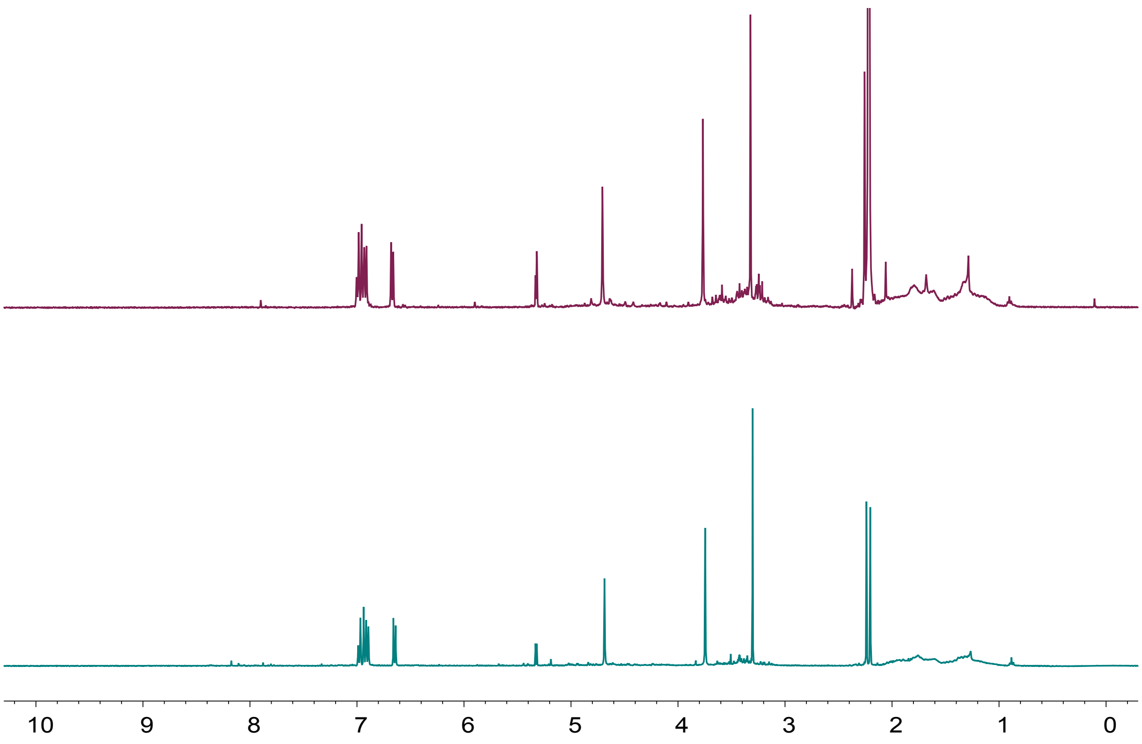


**Figure S24.** Comparison of $^1H$-NMR (400 MHz) 1b spectra in $CD_2Cl_2$ derived from precursors 1a (below) and 3a (above)

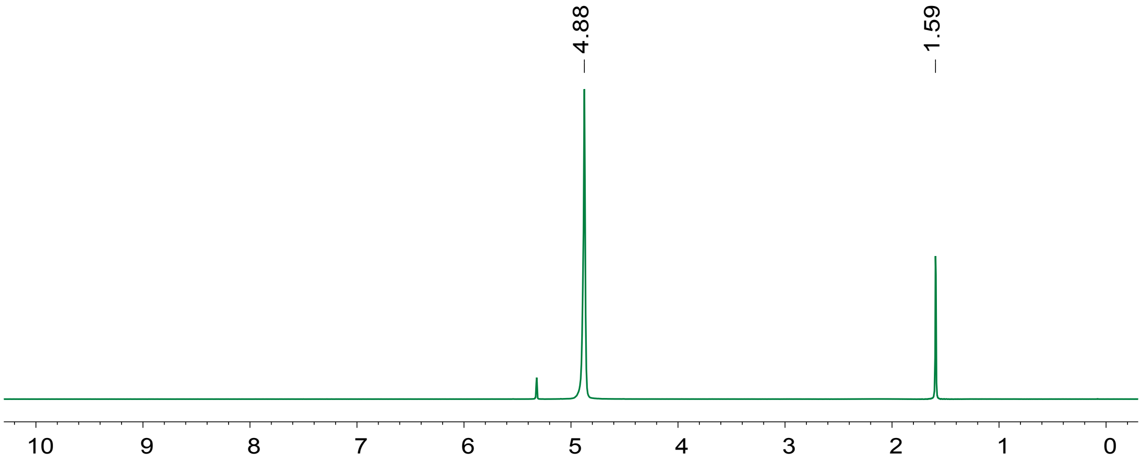


**Figure S25.** $^1H$-NMR (400 MHz) in $CD_2Cl_2$ of reaction medium of preliminary experiment with NaOH and DCM. Peak at 4.88 is primarily assigned to methanediol and peak at 1.59 belongs to water from NaOH and methanediol dehydration.

## 4. References


1. Archer, R. H.; Carpenter, J. R.; Hwang, S.-J.; Burton, A. W.; Chen, C.-Y.; Zones, S. I.; Davis, M. E., Physicochemical Properties and Catalytic Behavior of the Molecular Sieve SSZ-70. *Chem. Mater.* **2010**, *22*, 2563-2572.
2. Márquez, A.; Ávila, E.; Urbaneja, C.; Álvarez, E.; Palma, P.; Cámpora, J., Copper(I) Complexes of Zwitterionic Imidazolium-2-Amidinates, a Promising Class of Electroneutral, Amidinate-Type Ligands. *Inorg. Chem.* **2015**, *54*, 11007-11017.
3. Sánchez-Roa, D.; Santiago, T. G.; Fernández-Millán, M.; Cuenca, T.; Palma, P.; Cámpora, J.; Mosquera, M. E. G., Interaction of an imidazolium-2-amidinate (NHC-CDI) zwitterion with zinc dichloride in dichloromethane: role as ligands and C–Cl activation promoters. *Chem. Commun.* **2018**, *54*, 12586-12589.
